# In Situ Pseudo‐Halide Diffusion Enables Buried Interface Regulation and Crystallinity Enhancement in Perovskite Solar Cells

**DOI:** 10.1002/advs.75864

**Published:** 2026-05-29

**Authors:** Chao Gao, Li He, Changjiang Li, Long Fang, Wenzhong Shen

**Affiliations:** ^1^ College of Chemistry and Chemical Engineering Huangshan University Huangshan P. R. China; ^2^ Key Laboratory of Functional Materials Physics and Chemistry of the Ministry of Education Jilin Normal University Changchun P. R. China; ^3^ Institute of Solar Energy, and Key Laboratory of Artificial Structures and Quantum Control (Ministry of Education) School of Physics and Astronomy Shanghai Jiao Tong University Shanghai P. R. China; ^4^ Renewable Energy School Inner Mongolia University of Technology Ordos P. R. China

**Keywords:** buried interface engineering, high‐voltage perovskite solar cells, lithium formate polymorphs, pseudo‐halide diffusion, strain relaxation

## Abstract

Regulating buried interfaces is pivotal for suppressing interfacial defects and facilitating crystallization toward compact, high‐quality perovskite films. Here, we propose a thermally activated lithium formate (LiHCOO) buried interface strategy that triggers in situ pseudo‐halide diffusion, thereby simultaneously reconstructing the SnO_2_/perovskite interface and regulating perovskite nucleation and growth. High‐temperature treatment transforms LiHCOO from its low‐temperature hexagonal polymorph to a metastable monoclinic phase with a more open molecular packing structure, thereby enabling deeper HCOO^−^ diffusion into the buried perovskite. Diffused HCOO^−^ strongly interacts with undercoordinated Pb^2+^ sites, inhibiting pinhole formation, reducing trap density, compensating for halide vacancy related defects, and releasing residual tensile strain. Meanwhile, LiHCOO modification passivates the SnO_2_ surface by reducing oxygen vacancies and hydroxyl defects, improving interfacial electrical properties. This enhances the built‐in potential of perovskite devices from 0.91 to 1.00 V while optimizing energy level alignment. Ultimately, the optimized perovskite solar cell achieved a champion efficiency of 25.48%, with an open‐circuit voltage of 1.213 V and a fill factor of 82.57%, while also demonstrating outstanding long‐term stability. This work reveals polymorph‐mediated pseudo‐halide diffusion as a novel approach to low‐loss and robust perovskite photovoltaics.

## Graphical abstract

Thermally activated LiHCOO induces in situ pseudo‐halide diffusion, promoting buried interface strain release and perovskite crystallization. The monoclinic LiHCOO phase forms an open framework structure that enhances HCOO^−^ diffusion and drives interfacial restructuring. These processes optimize energy level alignment, suppress non‐radiative recombination, and accelerate carrier transport, yielding a high *V*_OC_ of 1.213 V and a PCE of 25.48%.

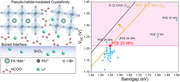

## 1 Introduction

Perovskite solar cells (PSCs) have developed rapidly as one of the representatives of next‐generation photovoltaic technology, with certified power conversion efficiencies (PCEs) exceeding 27%, approaching the level of the state‐of‐the‐art crystalline silicon heterojunction solar cells (certified PCE of 27.8%) [1, 2, 3, 4]. This breakthrough stems from the exceptional comprehensive optoelectronic properties of metal halide perovskite materials, primarily including high optical absorption coefficients, low exciton binding energies, long carrier diffusion lengths/lifetimes, and high defect tolerance [5, 6]. As the PCE of PSCs approaches its theoretical limit, the dual bottlenecks of efficiency degradation and structural instability have become increasingly prominent, posing critical challenges that must be overcome to advance their commercialization. Increasing evidence indicates that these bottlenecks originate not only from the top surface of the perovskite layer, but more critically from the buried interface directly contacting the electron transport layer (ETL) [7, 8, 9]. The buried interface dominates nucleation pathways, interfacial mechanical stresses, defect formation, band alignment, and early degradation chemical reactions [10, 11, 12, 13]. Due to the perovskite crystallization process initiating at this buried interface, its structural evolution and ion kinetics are inherently difficult to detect directly, making modulation extremely challenging. Therefore, developing engineering strategies for buried interfaces has become one of the core scientific directions for advancing PSC technology [14, 15, 16, 17, 18, 19].

In recent years, buried interface engineering has emerged as one of the most prominent research strategies for suppressing interface defects and enhancing PCE and stability of PSCs [18, 20, 21, 22]. Existing methods can be broadly categorized as follows: (i) Molecular bridging: Multifunctional small molecules (including sulfonates, carboxylates, and dipolar salts) simultaneously anchor to the ETL and perovskite lattice, forming chemically bonded charge transport pathways at the buried interface [23, 24, 25, 26, 27, 28, 29, 30, 31, 32]; (ii) Defect passivation: Achieved through Lewis base/acid additives, self‐assembled monolayers (SAMs) containing amines or boric acid, and sulfonate modifiers, which neutralize oxygen vacancies, Pb‐related trap states, and defects present in the perovskite bottom interface [33, 34, 35, 36]; (iii) Crystallization and strain regulation: Buried interface modifiers can regulate nucleation and grain growth kinetics, promote crystal orientation optimization, and construct stress‐relieving structures (e.g., organic networks and soft pseudo‐arch molecular frameworks) [31, 37, 38, 39]; and (iv) Interface phase engineering: Suppressing non‐radiative recombination and reducing lattice mismatch by introducing 2D Ruddlesden‐Popper layers and lattice‐matched oxides such as SrSnO_3_ [9, 40]. Despite these advances, many commonly used modifiers still primarily function as static passivation layers with limited coordination capabilities. They are unable to dynamically regulate buried interfaces during nucleation and grain growth, and thus rarely achieve simultaneous suppression of excessive nucleation, elimination of buried pinholes, control of crystal orientation, and release of tensile stress [15, 41]. Furthermore, the buried intermediate layer is often simplified as a homogeneous structure in studies; however, its intrinsic polymorphism and the arrangement of molecules at the SnO_2_/perovskite interface have yet to be thoroughly investigated. Many modifiers introduce undesirable effects, including reduced carrier mobility, increased optical parasitic absorption, and instability under continuous thermal or optical fields. Therefore, developing a novel buried interface strategy capable of dynamic reconstruction is crucial, as it is decisive for preparing structurally compact, pinhole‐free perovskite films with highly efficient carrier transport properties.

In this work, we report an in situ pseudo‐halide diffusion strategy enabled by the thermally induced polymorphic transformation of lithium formate (LiHCOO), which provides an effective route for simultaneously regulating buried interface chemistry, strain relaxation, and perovskite crystallization. High‐temperature activation converts LiHCOO into a monoclinic metastable phase featuring a more open and compliant molecular framework than the hexagonal phase. This flexible lattice promotes more extensive HCOO^−^ diffusion and stronger interactions with undercoordinated Pb^2+^ sites at the buried interface, leading to efficient passivation of deep‐level traps, effective compensation of halide vacancies, and pronounced suppression of non‐radiative recombination. The monoclinic phase enables superior interfacial reconstruction, resulting in more favorable band alignment, improved film crystallinity, and enhanced device performance. Accordingly, the PCE increases from 23.26% for the control device to 25.48% for the target device, with a stabilized PCE of 25.36%. The champion open‐circuit voltage (*V*_OC_) achieved in this study reaches 1.213 V. According to literature surveys, this value represents the highest reported *V*_OC_ for 1.55 eV bandgap perovskite absorbers employing buried interface engineering (Table S1 and Figure S1). Moreover, among the representative buried interface engineered PSCs included in our comparison with *V*_OC_ values higher than 1.213 V, our device exhibits the highest PCE. This corresponds to an exceptionally low voltage loss of 0.337 V and approaches ∼95% of the Shockley–Queisser (SQ) limit [42]. The LiHCOO‐modified buried interface exhibits outstanding ambient robustness, enabling unencapsulated devices to maintain 91.3% of initial PCE after 120 days of aging. Although other modifiers have also been explored for buried interface and crystallization engineering [43, 44, 45], this work focuses on the polymorphic LiHCOO system to reveal a distinct buried interface regulation mechanism. Here, the buried interlayer is not regarded merely as a passivation layer, but as a thermally responsive medium whose polymorphic transition governs diffusion kinetics and interfacial reconstruction during perovskite nucleation and growth. This study therefore, establishes polymorph‐mediated pseudo‐halide diffusion as a distinctive buried interface engineering strategy for advancing perovskite photovoltaics.

## 2 Results and Discussion

**FIGURE 1:**
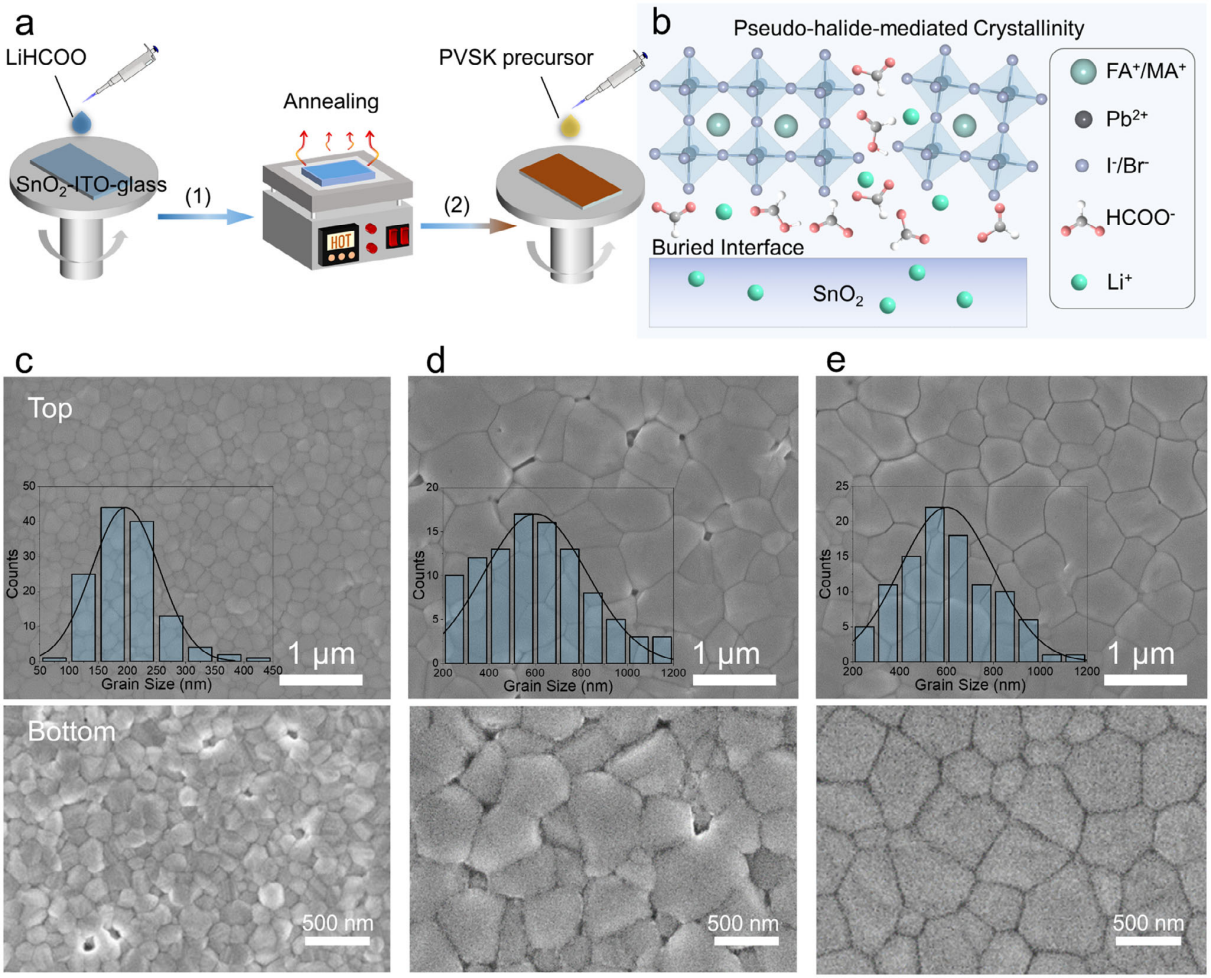
(a) Schematic illustration of the synthesis process for the LiHCOO buried interface in PSCs and (b) the diffusion behavior of Li^+^ and HCOO^−^. Top‐ and bottom‐view SEM images and grain size distributions of perovskite films: (c) without LiHCOO modification; (d) with LiHCOO modification annealed at 160°C; (e) with LiHCOO modification annealed at 320°C.

We propose a buried interface engineering that regulates perovskite crystallization kinetics by introducing lithium formate (LiHCOO) while simultaneously optimizing the interface contact between the perovskite and the electron transport layer (ETL). Specifically, a formate‐containing solution was spin‐coated onto the pre‐deposited SnO_2_ layer and thermally treated under a N_2_ atmosphere to form a LiHCOO buried interface, followed by deposition of the perovskite layer via a one‐step antisolvent process (Figure 1a). During annealing, formate anions (HCOO^−^) diffuse from the interlayer into the perovskite precursor medium and the buried perovskite region, where they coordinate with Pb^2+^ as pseudo‐halides to modulate nucleation and growth (Figure 1b) [39, 40]. Top‐view scanning electron microscopy (SEM) images reveal that the control film without LiHCOO modification exhibits a high nucleation density, resulting in ∼200 nm grains and distinct pinholes at the buried interface (Figure 1c). To analyze the morphology of the buried interface, we mechanically peeled the perovskite layer using an UV‐curable adhesive (Figure S2), thereby enabling SEM observation of this interface. As shown in Figure 1d, annealing the LiHCOO layer at 160°C resulted in an increase in perovskite grain size to ∼600 nm, indicating that nucleation density was effectively suppressed. However, significant pinholes remained present on the sample top surface and at buried interfaces under these conditions. In particular, by increasing the annealing temperature of the LiHCOO interlayer layer to 320°C, a dense and uniform perovskite film with a similarly large grain size (∼600 nm) was obtained, while completely eliminating pinhole defects (Figure 1e). This improvement likely stems from the enhanced activation of the LiHCOO interlayer, which strengthens chemical interactions at the SnO_2_/perovskite interface and promotes more controlled nucleation and subsequent crystal growth. These results demonstrate that high‐temperature activation of pseudo‐halide anion diffusion provides an effective approach for fabricating dense, large‐grain perovskite films with suppressed pinhole formation [46, 47, 48].

**FIGURE 2:**
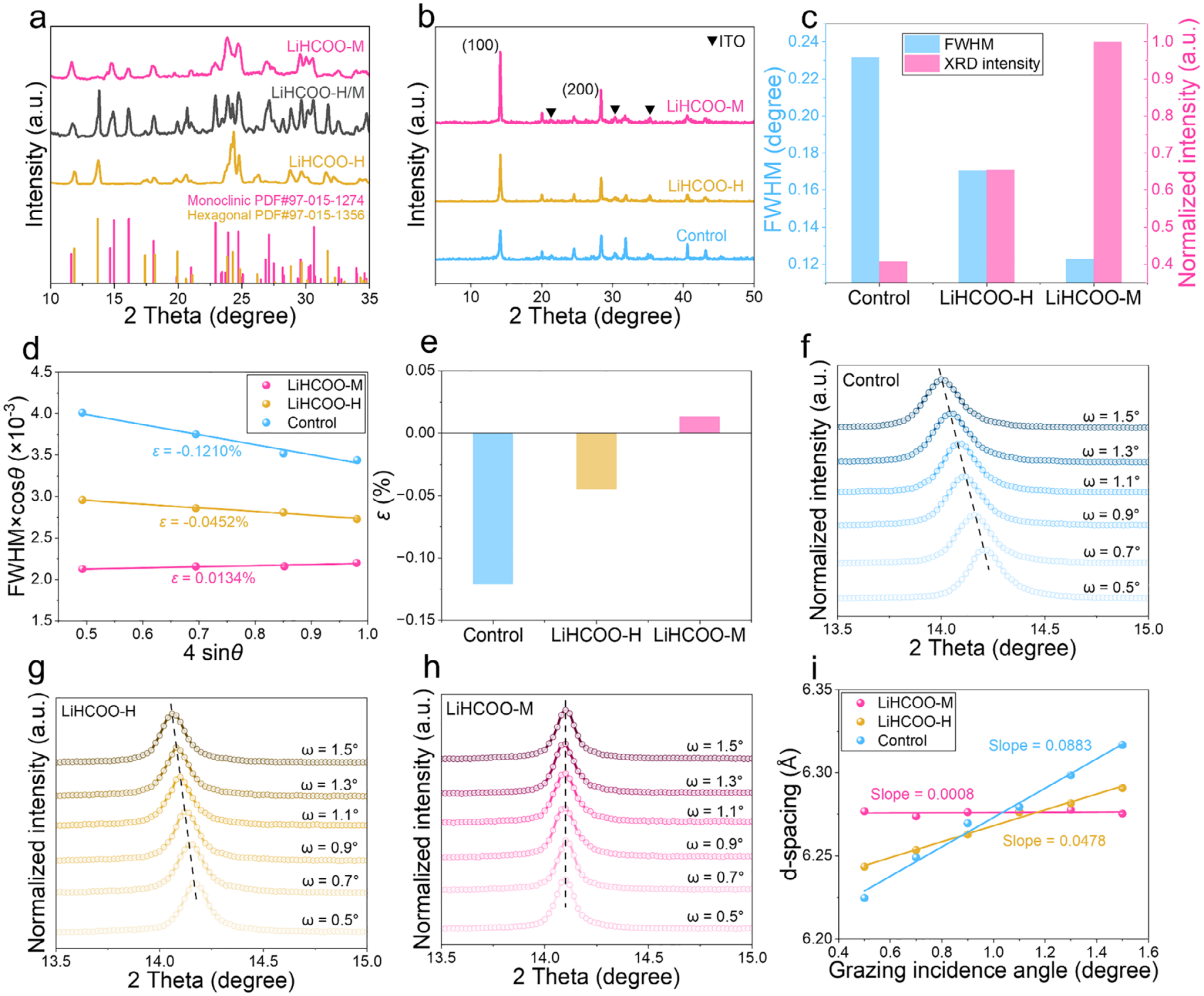
(a) XRD patterns of LiHCOO films with different crystalline phases (a.u., arbitrary units). (b) XRD patterns of perovskite films without LiHCOO modification and with LiHCOO‐H or LiHCOO‐M buried interface modification. (c) Comparison of the normalized intensity and FWHM of the (100) diffraction peak extracted from panel (b). (d) Williamson–Hall plots and (e) corresponding residual strain histograms of control, LiHCOO‐H‐modified, and LiHCOO‐M‐modified perovskite films. GIXRD spectra of perovskite films measured at different incident angles: (f) control, (g) LiHCOO‐H‐modified, and (h) LiHCOO‐M‐modified films. (i) Fitted curves of the d‐spacing values for the (100) planes as a function of incident angle.

To investigate how buried interface engineering is affected by thermally activated LiHCOO, we carried out systematic X‐ray diffraction (XRD) characterization on LiHCOO samples annealed at different temperatures. Figure S3 shows that the unannealed sample exhibits characteristic XRD peaks corresponding to the hydrated phase LiHCOO·H_2_O. As shown in Figure 2a, annealing at 120°C induces dehydration of LiHCOO·H_2_O and leads to the formation of the low‐temperature hexagonal polymorph (LiHCOO‐H, space group P6_3_) [49]. When the annealing temperature is further increased to 240°C, additional diffraction features emerge, indicating a mixed‐phase state composed of LiHCOO‐H and a monoclinic component (LiHCOO‐M, space group C2/c) [50]. After annealing at 320°C followed by rapid quenching under anhydrous and oxygen‐free conditions, the resulting XRD pattern is predominantly assignable to the monoclinic LiHCOO‐M phase, consistent with retention of a metastable high‐temperature structural state [49, 50]. This observation suggests that the rapid‐cooling process kinetically locks the lattice arrangement. The resulting temperature‐dependent structural evolution helps explain the distinct buried interface regulation effects observed under LiHCOO‐H and LiHCOO‐M conditions. Detailed molecular packing modes and lattice configurations for LiHCOO‐H and LiHCOO‐M are provided in Figure S4a,b. This temperature‐dependent polymorphic evolution plays a pivotal role in regulating the buried interface. Compared with the compact LiHCOO‐H phase, the LiHCOO‐M phase features a more loosely molecular packing arrangement, which facilitates the diffusion of formate anions during perovskite crystallization. The thermally activated diffusion in this work significantly reduces nucleation density and promotes the growth of compact perovskite grains, consistent with the improved SEM morphology. This behavior originates from the annealing‐induced polymorphic transition of LiHCOO, which creates a more open molecular packing structure and thereby facilitates ionic redistribution from the buried interlayer during perovskite crystallization.

By preparing perovskite films at three buried interfaces, we further explored how the polymorphic states of LiHCOO influence the perovskite crystallization process: the unmodified control substrate, the LiHCOO‐H interface, and the LiHCOO‐M interface. The XRD patterns in Figure 2b,c show that the control film exhibits the weakest intensity for the (100) diffraction peak, while LiHCOO‐H provides a moderate enhancement and LiHCOO‐M yields the strongest (100) peak intensity. Correspondingly, the full width at half maximum (FWHM) of the (100) peak progressively narrows from the control to the LiHCOO‐H sample and further to the LiHCOO‐M sample, indicating significantly improved crystallinity when the monoclinic phase is used as the buried interlayer. Residual strain is a critical factor that affects the efficiency and stability of PSCs, typically originating from external thermal stimuli and from thermal expansion mismatch at the buried interface [51, 52, 53]. Williamson–Hall analysis [54] (Figure 2d,e) shows that the perovskite film grown on LiHCOO‐M exhibits a remarkably low lattice strain of 0.0134%, which is substantially smaller than that of the LiHCOO‐H‐based film at −0.0452% and the control film at −0.1210%. Depth‐resolved grazing‐incidence XRD (GIXRD) further corroborates this trend (Figure 2f–h) [8, 55]. As the incident angle increases from 0.5° to 1.5°, the diffraction peaks of the control film gradually shift toward smaller angles, indicating tensile lattice expansion. This peak movement is less evident in the LiHCOO‐H‐based film and becomes negligible in the LiHCOO‐M‐based film, confirming that the monoclinic LiHCOO‐M layer effectively suppresses in‐plane tensile strain (Figure S5). As shown in Figure 2i, fitting the lattice spacing as a function of incident angle yields a decreasing slope from 0.0883 for the control film to 0.0478 for the LiHCOO‐H sample and eventually to only 0.0008 for the LiHCOO‐M sample, demonstrating nearly complete release of residual tensile strain. In contrast to the compact hexagonal phase, LiHCOO‐M exhibits a more open molecular packing structure, thereby facilitating broader HCOO^−^ redistribution from the buried interlayer into the perovskite film and enhancing its ionic interactions with undercoordinated Pb^2+^ sites [56, 57, 58]. These interactions not only compensate for local halide vacancies and optimize the Pb‐X (X = I/Br) coordination environment, but also effectively mitigate local stress concentration during film formation. Consequently, subsequent analyses will primarily focus on comparing the unmodified control sample with the LiHCOO‑M‐modified sample.

**FIGURE 3:**
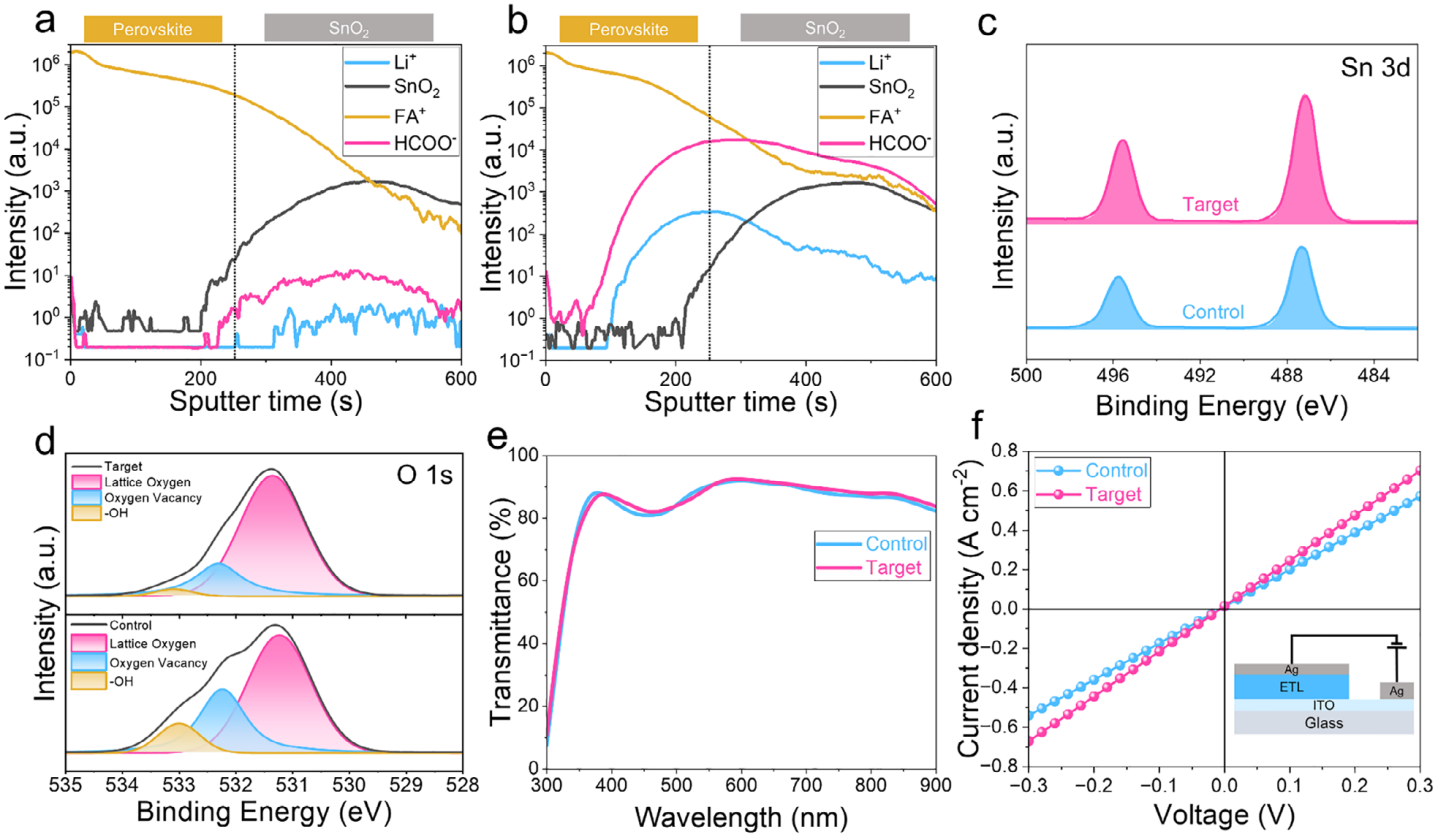
TOF‐SIMS depth profiles of perovskite films (a) without and (b) with LiHCOO buried interface modification. XPS spectra of SnO_2_ films: (c) Sn 3d (d) O 1s states with and without LiHCOO modification. (e) Optical transmittance spectra and (f) electrical conductivity curves of SnO_2_ substrates, with the inset showing a schematic of the tested structure.

We investigated the ion diffusion processes in the control and target samples using time‐of‐flight secondary ion mass spectrometry (TOF‐SIMS), as shown in Figure 3a,b. The depth profiles obtained from TOF‐SIMS (Figure S6a,b) reveal pronounced enrichment of Li^+^ and HCOO^−^ at the SnO_2_/perovskite interface in the target sample. The signal intensities in the target film are several orders of magnitude higher than those in the control film, providing clear evidence for the diffusion of HCOO^−^ into the perovskite film. Although part of the detected Li^+^ and HCOO^−^ signals may arise from residual ions in the analysis chamber, this background contribution does not alter the conclusion, as the concentrations of Li^+^ and HCOO^−^ at the buried interface in the target sample remain approximately three orders of magnitude higher than those in the control sample [59, 60]. Notably, the TOF‐SIMS curves of the LiHCOO‐H sample exhibit a clear contraction in the Li^+^ and HCOO^−^ signal distribution compared with the LiHCOO‐M sample (Figure S7a–d). This directly indicates that ion diffusion in the LiHCOO‐H system is significantly restricted. Such behavior is consistent with the inherently tighter molecular packing of the LiHCOO‐H lattice, which impedes long‐range ion diffusion. In contrast, the target sample based on the LiHCOO‐M interlayer displays a broad and deep penetration of Li^+^ and HCOO^−^, demonstrating that the monoclinic phase enables more efficient diffusion pathways in the perovskite layer. Li^+^ and HCOO^−^ exhibit no obvious difference in diffusion direction, whereas a slight discrepancy in diffusion depth is observed, likely due to their distinct chemical interactions at the buried interface. The spatial distribution of Li^+^ and HCOO^−^ indicates that formate‐driven interfacial modification plays a crucial role in promoting uniform nucleation, relaxing local strains, and enabling the formation of dense, highly crystalline perovskite films. Since the formation of the monoclinic phase in the target sample requires annealing at 320°C, we first investigated whether this high‐temperature treatment would affect the structural characteristics of SnO_2_. XRD measurements show that SnO_2_ films annealed at 320°C display diffraction patterns that are nearly identical to those of SnO_2_ films annealed at 160°C, which is the typical processing temperature for SnO_2_ (Figure S8). Through X‐ray photoelectron spectroscopy (XPS) analysis, the interfacial chemical interactions between LiHCOO and SnO_2_ were investigated. Compared to the control sample, the Sn 3d peak binding energy shifted negatively and the O 1s peak shifted positively in target LiHCOO‐M‐modified SnO_2_ (Figure 3c,d). Deconvolution analysis of the O 1s spectrum revealed three components: lattice oxygen, oxygen vacancies, and surface hydroxyls (─OH) [61, 62]. The latter serve as active centers inducing the perovskite phase transition. After LiHCOO‐M modification, the contents of oxygen vacancies and surface hydroxyls decreased significantly (by 30.79% (from 29.65% to 20.52%) and 74.44% (from 9.90% to 2.53%), respectively), indicating that defects were effectively suppressed, thereby reducing interfacial recombination and enhancing charge extraction capability. Optical transmission spectra reveal that the transmittance of the SnO_2_ substrate remains essentially unchanged within the primary perovskite absorption range of 400–800 nm after modification with LiHCOO (Figure 3e), confirming the absence of additional parasitic optical losses. Furthermore, LiHCOO doping significantly enhances the electronic properties of the ETL. Using ITO/SnO_2_/LiHCOO‐M (with or without)/Ag structures, we find that the electrical conductivity of the LiHCOO‐M‐modified SnO_2_ layer increases by ∼1.23 times compared with the control sample (Figure 3f). This improvement demonstrates that lithium doping optimizes the electrical properties of SnO_2_, enhancing electron transport capacity at the SnO_2_/perovskite interface. The coordinated enhancement of conductivity and suppression of defects enables the fabrication of PSCs with higher *V*_OC_ and fill factor (FF), further validating the pivotal role of LiHCOO‐M modification in high‐performance PSCs. It should be noted that Li^+^ and HCOO^−^ play distinct yet cooperative roles in this system. Li^+^ mainly contributes to the electrical optimization of the SnO_2_ side of the buried interface, whereas HCOO^−^ is more directly involved in regulating crystallization and passivating defects in the perovskite.

**FIGURE 4:**
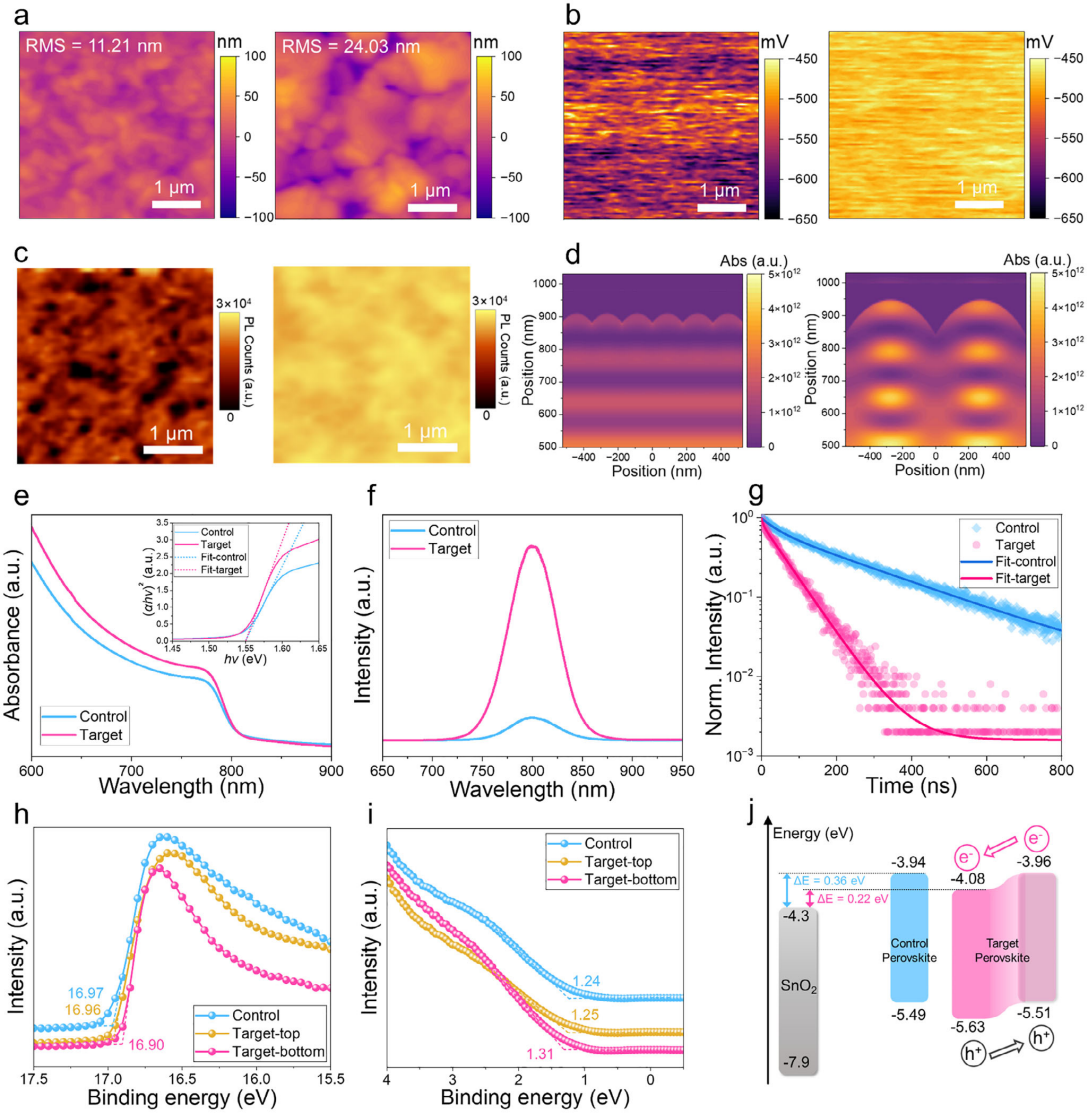
(a) AFM images of perovskite films without (left) and with (right) LiHCOO modification. (b) Surface potential maps obtained by KPFM for the control (left) and the target (right) samples. (c) Top‐view wide‐field hyperspectral PL mapping images of the control (left) and the target (right) samples. (d) Simulated optical absorption distribution maps at *λ* = 680 nm for the control (left) and the target (right) samples. (e) UV–vis absorption spectra, with the inset showing the bandgap fitting using the Tauc method. (f) Steady‐state PL and (g) TRPL spectra of the control and target samples. (h, i) UPS spectra of the control and target samples, and (j) the schematic illustration of the band structure.

We examined how LiHCOO modifies the surface morphology of perovskite films by conducting atomic force microscopy (AFM) measurements on both the control and target samples. The root mean square (RMS) roughness of the control sample is 11.21 nm, whereas that of the target sample increases to 24.03 nm (Figure 4a). The AFM height profiles shown in Figure S9a–f further confirm that the target sample exhibits a more pronounced topography. The increased roughness is attributed to the larger perovskite grains and enhanced vertical crystal growth induced by the LiHCOO‐M buried interface modification, which produces more prominent surface undulations. Despite this higher surface roughness, Kelvin probe force microscopy (KPFM) measurements (Figure 4b) reveal that the target sample exhibits a higher and more uniform surface electronic chemical potential (ECP). The control sample displays a surface potential variation more than three times larger than that of the target sample (Figure S10a–f), indicating pronounced electronic inhomogeneity. The improved uniformity of ECP in the target film is likely associated with its larger grain size and higher crystallinity, resulting in fewer grain‐boundary fluctuations in local electronic structure and more favorable pathways for charge transport. Wide‐field hyperspectral photoluminescence (PL) mapping (Figure 4c) further supports the beneficial impact of buried interface engineering. The target sample displays significantly higher PL emission intensity and a more homogeneous emission profile compared with the control film. In contrast, the control sample shows distinct bright and dark regions, where the dark domains correspond to areas with higher non‐radiative recombination, reflecting the presence of trap‐rich regions. The spatially uniform PL response in the target sample can be attributed to the improved crystallinity and uniformity achieved by the LiHCOO‐M modification.

To gain deeper insight into the optical consequences of the modified surface topography, we performed finite‐difference time‐domain (FDTD) simulations following established modeling approaches (Note S1) [63, 64, 65]. As shown in Figure 4d, the target sample exhibits stronger optical absorption compared with the control sample. Furthermore, regions with surface undulations display distinct resonance‐enhanced absorption bands, and cross‐sectional views of the 3D model reveal a pronounced moth‐eye effect [66, 67, 68]. This enhancement phenomenon arises from the scattering of Poynting vectors by larger grains and enhanced surface texture within the target film, thereby inducing Mie‐type resonant scattering (Figure S11) [69]. Such scattering increases the effective optical path within the perovskite layer (Figure S12), thereby promoting stronger photon coupling and more efficient light absorption in the perovskite active layer.

Consistent with FDTD simulation results, the UV–vis spectra reveal that the target sample exhibits significantly higher absorption intensity than the control film in the short‐wavelength range (Figure 4e). Fitting results obtained using the Tauc method indicate that both films retain the same optical bandgap (1.55 eV). This confirms that LiHCOO modification enhances light utilization efficiency without altering the intrinsic bandgap of the perovskite absorber layer [70]. Steady‐state photoluminescence (PL) spectra further demonstrate that the target film displays a markedly stronger emission intensity than the control sample (Figure 4f), which is consistent with the PL mapping results. This enhancement indicates suppressed non‐radiative recombination, reduced trap‐state density, and improved crystalline quality arising from the LiHCOO‐M buried interface modification. However, for the complete PSC architecture, the target cell exhibits a lower PL intensity than the control cell, which is attributed to more efficient carrier transport in the target device (Figure S13). To investigate the carrier dynamics, time‐resolved photoluminescence (TRPL) measurements were performed on SnO_2_‐quenched perovskite devices (Figure 4g). Bi‐exponential fitting (Note S2) shows that the average carrier lifetime (*τ*_avg_) decreases from 244.86 ns in the control sample to 62.28 ns in the target film (Table S2). This faster PL decay is characteristic of more efficient interfacial charge extraction, indicating that the modified SnO_2_/perovskite interface in the target sample possesses a stronger interfacial built‐in field.

The quenching enhancement effect of free carriers can be verified by ultraviolet photoelectron spectroscopy (UPS) (Figure 4h,i). The Fermi level (*E*_F_) and valence band maximum (VBM) of the control film are −4.25 and −5.49 eV (Table S3), respectively, yielding a conduction band minimum (CBM) of −3.94 eV when combined with the 1.55 eV optical bandgap (Tauc method). For the top surface of the target sample, the calculated values for *E*_F_, VBM, and CBM are −4.26, −5.51, and −3.96 eV, respectively. Ion‐beam etching reveals even deeper band positions at the perovskite bottom buried interface, with *E*_F_, VBM, and CBM values of −4.32, −5.63, and −4.08 eV, respectively. These shifts indicate that LiHCOO modification adjusts the perovskite's *E*_F_ toward more favorable alignment with SnO_2_, thereby reinforcing the interfacial electric field and enabling higher *V*_OC_. The resulting energy level diagram (Figure 4j) shows that the energy offset between the perovskite and SnO_2_ conduction bands is reduced from 0.36 eV in the control film to 0.22 eV in the target film. The monoclinic LiHCOO‐M phase induces an interfacial dipole at the SnO_2_/perovskite interface. Through dipole‐driven energy level alignment, it modulates the band structure, reduces the interfacial energy barrier, establishes a more optimal energy gradient, and forms a quasi‐bulk heterojunction. These characteristics collectively enhance carrier transport ability, ultimately improving PSC performance.

**FIGURE 5:**
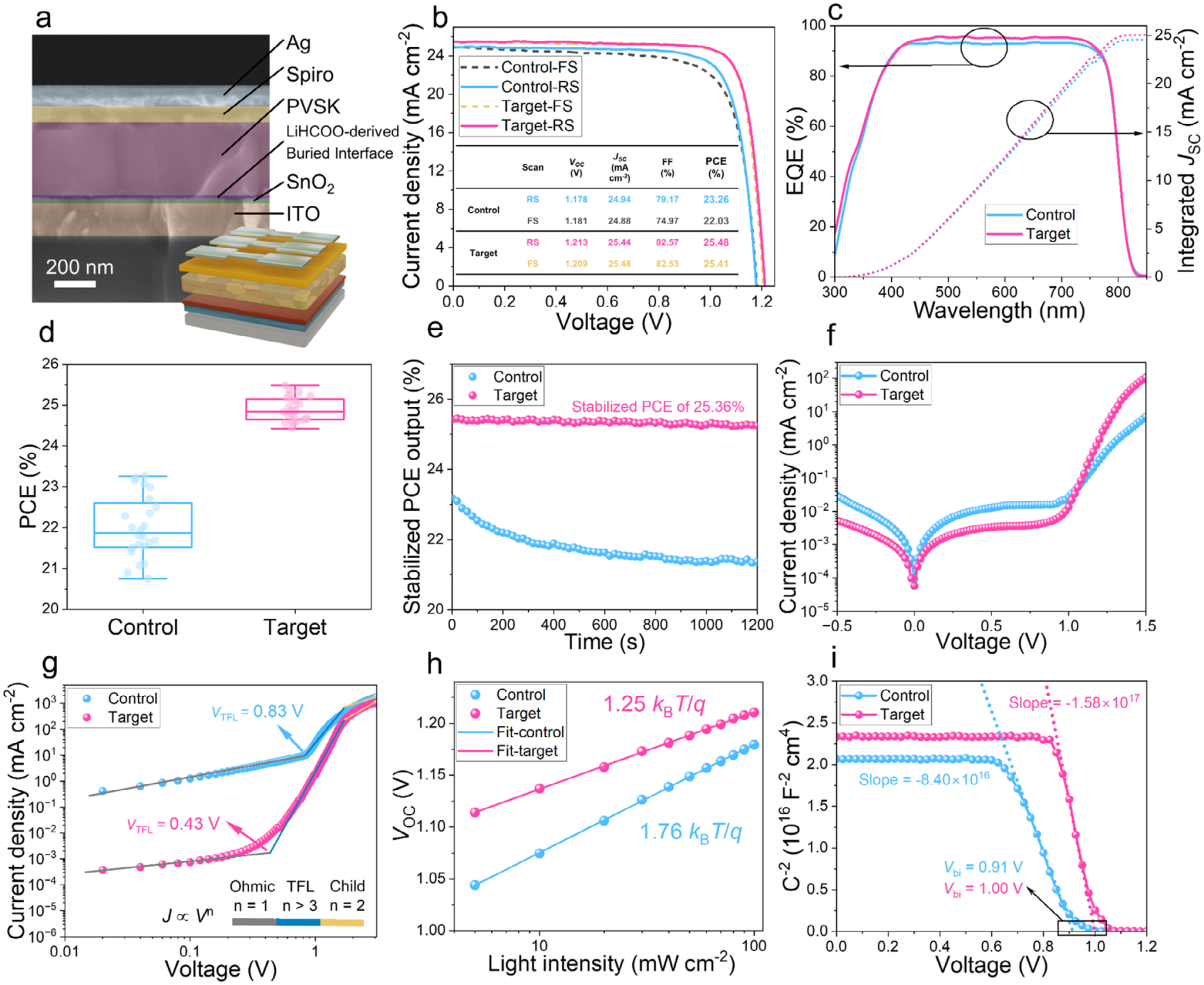
(a) Cross‐sectional SEM image of the PSC based on a LiHCOO‐derived buried interface, with the inset showing the schematic device architecture, Spiro: 2,2′7,7′‐tetrakis‐(N,N‐di‐p‐methoxyphenyl‐amine)‐9,9′‐spirobifluorene, PVSK: perovskite. (b) *J–V* characteristics of the control and target devices measured in both forward scan (FS) and reverse scan (RS) modes. (c) EQE spectra and integrated *J*_SC_ of the corresponding devices. (d) Statistical distribution of PCEs. (e) Steady‐state PCE output under continuous illumination. (f) Dark *J–V* curves of the PSCs. (g) SCLC curves of the electron‐only devices. (h) Dependence of *V*_OC_ on light intensity. (i) Mott–Schottky plots of the control and target devices.

Furthermore, regular n‐i‐p PSCs were fabricated using a LiHCOO‐derived buried interface, as shown in Figure 5a. The cross‐sectional SEM image presents the overall multilayer device architecture. The buried interfacial region is formed from the initial LiHCOO treatment, rather than remaining as an intact LiHCOO layer after thermal activation and perovskite deposition. Notably, the perovskite film grown on the LiHCOO‐derived buried interface exhibits highly vertical grain alignment, in contrast to the control sample, which shows pronounced grain boundaries and discontinuous domains (Figure S14). These abundant grain boundaries in the control device introduce trap‐rich regions that hinder charge transport along the out‐of‐plane direction. The photovoltaic characteristics, measured under standard AM 1.5G illumination with a 0.046 cm^2^ aperture, are presented in Figure 5b. The LiHCOO‐modified PSC delivers a significantly enhanced PCE, increasing from 23.26% for the control device to 25.48% (reverse scan (RS)). Specifically, the control PSC exhibits a *V*_OC_ of 1.178 V, a (short‐circuit current density) *J*_SC_ of 24.94 mA cm^−2^, and a FF of 79.17%. In comparison, the target device achieves an elevated *V*_OC_ of 1.213 V, an increased *J*_SC_ of 25.44 mA cm^−2^, and a boosted FF of 82.57%. Through calculation of the hysteresis index (HI = (PCE_reverse_ − PCE_forward_) / PCE_reverse_), we observed a distinct forward‐reverse difference in the control device, with an absolute difference in forward and reverse FFs reaching 4.2% (HI = 0.0529). This indicates significant ion migration occurring within the PSCs. In comparison, the LiHCOO‐modified device displays nearly identical forward and reverse characteristics (HI = 0.0027). In the target device, the suppression of hysteresis can be attributed to improved perovskite crystallization achieved through LiHCOO diffusion and a reduction in interfacial ionic defects. The improvement in device performance brought by LiHCOO modification is further confirmed by external quantum efficiency (EQE) measurements. As shown in Figure 5c, the target device exhibits an overall enhancement in EQE. This enhancement effect originates from the large grains and roughened perovskite morphology induced by LiHCOO diffusion, which produces beneficial light scattering and anti‐reflection effects. As a result, the integrated *J*_SC_ of EQE increases from 24.56 to 25.05 mA cm^−2^, which closely matches the improvement observed in the *J‐V* curves. Analysis of the first derivative of the EQE curves (Figure S15) reveals that the FA‐dominant perovskite material used in this study exhibits a device bandgap of ∼1.55 eV, consistent with the optical bandgap obtained via the Tauc fitting method. Apart from the enhancement in *J*_SC_, the target device exhibits significantly improved *V*_OC_ and FF compared to the control cell. This improvement primarily stems from the optimized energy level alignment at the perovskite interface with LiHCOO modification and the significant reduction in non‐radiative recombination losses at the buried interface. The champion target device exhibits a *V*_OC_ of 1.213 V, corresponding to a voltage loss of only 0.337 V. To further elucidate the origin of *V*_OC_ losses, we analyzed the quasi‐Fermi level splitting (QFLS) of the control and target samples. Based on the detailed balance model, the radiative limit of the *V*_OC_ (*V*_OC, rad_) was calculated using the following expression [71, 72]:

$$\begin{array}{ccc} V_{\mathrm{OC},\;\mathrm{rad}\;} & = & \frac{k_{B}T}{q}\;ln\left(\frac{\int \mathrm{EQ}E_{\mathrm{PV}}\left(E\right)\times \phi_{AM1.5}\left(E\right)dE}{\int \mathrm{EQ}E_{\mathrm{PV}}\left(E\right)\times \phi_{BB}\left(E\right)dE}+1\right)\\ & = & \frac{k_{B}T}{q}\;\ln\left(\frac{J_{\mathrm{SC}}}{J_{0}^{\mathrm{rad}\;}}+1\right) \end{array}$$

where the *ϕ_AM1.5_*, *ϕ_BB_*, and *J*_0_^rad^ correspond to the AM 1.5G solar spectrum, the black‐body radiation spectrum, and the radiative dark recombination current, respectively. The calculated *V*_OC, rad_ for the target sample is 1.265 V (Figure S16). The QFLS is then obtained using the following relation:

$$\mathrm{QFLS}\;=qV_{\mathrm{oc},\mathrm{rad}}+k_{B}T\ln\left(\mathrm{PLQY}\right)$$

We evaluated Δ(*qV*_OC, rad_ − QFLS), which decreases from 76 meV in the control sample to 48 meV in the target sample, indicating a significant reduction in energy loss [71, 73]. Based on the QFLS analysis, we attribute the remarkably low *V*_OC_ loss in the target device to the effective suppression of non‐radiative recombination and the improved energy level alignment at the SnO_2_/perovskite interface.

We fabricated a series of devices incorporating different concentrations of LiHCOO, as shown in Figure S17a,b. The results reveal a rise‐and‐fall trend in PCE with increasing concentration, with the optimal performance obtained at 15 mg mL^−1^. Beyond this concentration dependence, we also examined the influence of LiHCOO polymorphism on device performance (Figure S18a,b and Table S4). As the buried interlayer transitions from the LiHCOO‐H phase to the LiHCOO‐M phase, the PCE increases progressively and the hysteresis behavior is significantly suppressed. As shown in Figure 5d, statistical results indicate that the LiHCOO‐M modified device exhibits a higher overall level of PCE. Under continuous AM 1.5G illumination conditions, the control device exhibited pronounced PCE decay within the initial 20 min, whereas the target device maintained a stable PCE of 25.36% (Figure 5e). This rapid initial degradation of the control PSC likely reflects an early‐stage burn‐in process associated with its inferior buried interface quality [74, 75]. In contrast, LiHCOO modification suppresses interfacial defects, thereby stabilizing charge extraction and reducing early‐stage performance loss. This demonstrates the improved operational stability enabled by the buried interface design based on pseudo‐halide anion regulation.

Dark *J–V* measurements show that the target device exhibits a markedly reduced current under reverse bias, indicating effective suppression of leakage currents and a corresponding reduction in defect‐mediated conductive pathways (Figure 5f). In the forward‐bias regime from 1.1 to 1.5 V, the target device shows higher injection current than the control, confirming that LiHCOO interface modification substantially lowers the injection barrier between SnO_2_ and the perovskite layer. This observation is fully consistent with the UPS results and further verifies the enhanced charge‐transport capability.

Additionally, we fabricated ETL‐only devices and studied the effects of LiHCOO modification on the trap‐state density and recombination characteristics of perovskites using the space‐charge‐limited current (SCLC) method (ITO/SnO_2_/LiHCOO/Perovskite/PCBM/Ag, where PCBM is [6,6]‐phenyl‐C61‐butyric acid methyl ester) (Figure 5g). SCLC results demonstrate that diffusion‐induced perovskite crystallization at the buried interface via LiHCOO‐M significantly suppresses charge recombination. The trap‐filled limit voltage (*V*_TFL_) of the control device is 0.83 V, whereas that of the target device is 0.43 V. According to the equation:

$$V_{\mathrm{TFL}}=\frac{qN_{t}L^{2}}{2\varepsilon_{0}\varepsilon_{r}}$$

where *q* is the charge, *N*_t_ is the trap‐state density, *L* is the perovskite thickness, *ε_0_* is the vacuum permittivity, and *ε_r_* is the relative permittivity [76, 77]. The calculated electron trap densities for the control and target devices are 1.83 × 10^16^ and 9.51 × 10^15^ cm^−3^, respectively. These results confirm that LiHCOO modification yields a perovskite layer with a remarkably low defect‐state density.

We investigated the carrier recombination mechanism in the device by analyzing the relationship between the *V*_OC_ and the incident light intensity (Φ) (Figure 5h). The slope is determined by the following expression:

$$\;\Delta V_{\mathrm{OC}}=\frac{nk_{B}T\Delta \ln\Phi }{q}$$

where *n* is the ideality factor, *k*_B_ is the Boltzmann constant and T is the temperature. This equation can evaluate the extent of trap‐assisted Shockley–Read–Hall (SRH) recombination [78, 79, 80]. The control device exhibits a slope of 1.76 *k*_B_T/q, while the LiHCOO‐modified device exhibits a substantially reduced slope of 1.25 *k*_B_T/q. This reduction indicates a significant suppression of trap‐assisted Shockley–Read–Hall recombination after buried interface engineering. These findings align with the conclusions drawn from SCLS analysis, confirming that the defect density is reduced in the LiHCOO‐modified perovskite films. The improvement in the ideality factor is associated with the interaction between HCOO^−^ and undercoordinated Pb^2+^, which passivates iodine vacancies [39]. To substantiate this mechanism, first‐principles calculations were performed for iodine‐vacancy configurations with relatively low formation energies (Figure S19a,b). The incorporation of HCOO^−^ enlarges the surface Pb─I─Pb bond angle from 145.26° to 153.45°, consistent with the XRD‐derived enhancement in crystallographic ordering. This transformation is driven by the strong interaction between the HCOO^−^ pseudo‐halide anion and Pb^2+^, which alleviates octahedral distortion after iodine atom removal, thereby stabilizing a more symmetrical cubic crystal lattice.

Between the SQ‐limit and the experimentally measured FF, FF loss comprises two components: non‐radiative loss and charge transport loss. According to the detailed balance theory, the SQ‐limit FF for perovskites with a bandgap of 1.55 eV is 90.2% (Figure S20). In the absence of charge transport losses, the maximum achievable FF can be determined using the following equation [81]:

$$\mathrm{FF}=\frac{V_{\mathrm{OC}}-\frac{k_{B}T}{q}\ln\left(\frac{qV_{\mathrm{OC}}}{k_{B}T}+0.72\right)}{V_{\mathrm{OC}}+\frac{k_{B}T}{q}}$$

The enhancement in FF achieved by the LiHCOO buried interface modification arises from the suppression of both charge‐transport losses and non‐radiative losses. Based on the Mott–Schottky analysis, we further investigated the impact of LiHCOO on the device electrical properties (Figure 5i). The built‐in potential (*V*_bi_) at the SnO_2_/perovskite depletion region increases from 0.91 V in the control device to 1.00 V in the target device, indicating an enhanced built‐in field after LiHCOO modification. In addition, the carrier density (*N*_D_) at the SnO_2_/perovskite interface in the target device is only 0.53 times that of the control device (Note S3), suggesting mitigated interfacial charge accumulation at the buried interface. To further support this conclusion, dark electrochemical impedance spectroscopy (EIS) measurements were performed, and the corresponding Nyquist plots are shown in Figure S21. The LiHCOO‐modified device exhibits a higher recombination resistance (*R*_rec_) of 261.8 kΩ, compared with 71.9 kΩ for the control device, indicating suppressed interfacial recombination and reduced carrier accumulation. These results collectively confirm that LiHCOO modification improves the interfacial electrical properties and suppresses interfacial recombination at the SnO_2_/perovskite buried interface, thereby contributing to the enhanced *V*_OC_ and FF.

**FIGURE 6:**
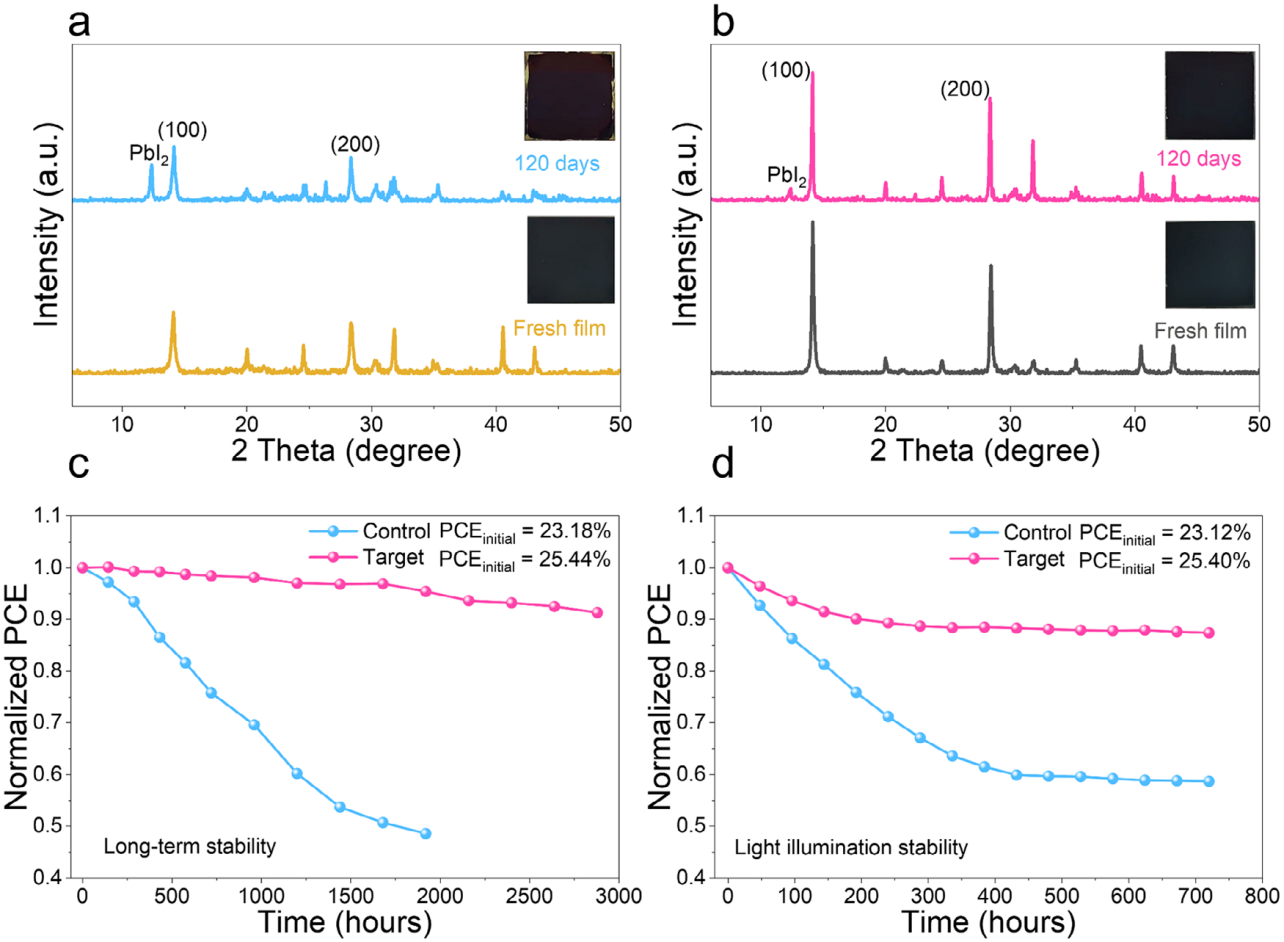
XRD patterns of perovskite films for (a) the control and (b) the target samples before and after aging for 120 days under ambient conditions. The insets show the corresponding perovskite film images. (c) Long‐term stability of the devices tested under ∼20% RH and 25°C in air. (d) Light stability of the devices under continuous 1 sun illumination.

Long‐term stability is a vital criterion for evaluating the commercial viability of PSC technology [82, 83]. To explore the robustness of the LiHCOO‐modified buried interface under demanding environmental conditions, we examined the structural and device‐level stability of the perovskite films during extended exposure. As shown in Figure 6a,b, XRD patterns collected after 120 days reveal a pronounced PbI_2_ diffraction peak in the control film, while the peak is significantly suppressed in the target film (Figure S22). After long‐term exposure to ambient air, the unprotected control perovskite film undergoes severe degradation accompanied by hydration and decomposition, resulting in visible discoloration and the formation of yellow PbI_2_. In contrast, the target film undergoes negligible color change and exhibits a substantially attenuated PbI_2_ signature. The normalized PCE evolution presented in Figure 6c further highlights this difference. After 120 days in ambient conditions (temperature: 25°C, relative humidity (RH): ∼20%), the unencapsulated LiHCOO‐modified device still retains 91.3% of its initial PCE of 25.44%. Meanwhile, the unencapsulated control device retains only 48.6% of its initial PCE of 23.18% after 80 days of aging. The markedly improved stability arises from LiHCOO‐induced buried interface protection, which enhances perovskite crystallinity, suppresses undercoordinated defect sites, and strengthens resistance to moisture‐driven degradation. This synergistic stabilization is clearly reflected in the illumination stability results shown in Figure 6d. After 720 h of continuous light exposure, the target device maintains 87.4% of its initial PCE of 25.40%, while the control device decreases to 58.7% of its initial PCE of 23.12%. To further evaluate the operational stability of the devices, maximum power point (MPP) tracking was performed on unencapsulated PSCs under continuous 1 sun illumination at 65°C and ∼50% RH (Figure S23). The control device decayed to below 50% of its initial PCE after 480 h, whereas the target device still retained 86.4% of its initial PCE after the same aging period, further confirming the substantially enhanced operational robustness enabled by LiHCOO buried interface engineering. We further validated the enhanced stability and assessed the surface hydrophobicity by performing water contact angle measurements (Figure S24). The contact angles for the control and target perovskite films are 42.9° and 89.5°, respectively, confirming that the target film exhibits significantly superior hydrophobic characteristics. The pseudo‐halide‐mediated crystallization regulation method can significantly suppress the formation of moisture‐sensitive defect sites, thereby leading to new routes for high‐stability PSCs.

## 3 Conclusion

In summary, we establish a thermally activated LiHCOO‐enabled in situ pseudo‐halide diffusion strategy that concurrently reconstructs the SnO_2_/perovskite buried interface and directs perovskite crystallization. The temperature‐driven polymorphic transition from the low‐temperature hexagonal phase to a metastable monoclinic phase creates a more open and compliant lattice, enabling deep and efficient HCOO^−^ transport into the perovskite layer. This polymorph‐assisted diffusion suppresses excessive nucleation, releases residual tensile strain from −0.1210% in the control film to 0.0134% in the LiHCOO‐M sample, and yields compact, pinhole‐free, large‐grained perovskite films. Meanwhile, the reconstructed buried interface strengthens interactions with SnO_2_, suppressing oxygen‐vacancy content by 30.79% and hydroxyl‐related defects by 74.44%, thereby enhancing carrier extraction. These synergistic improvements optimize band alignment, increase the built‐in potential from 0.91 to 1.00 V, and suppress non‐radiative recombination, enabling a *V*_OC_ enhancement from 1.178 to 1.213 V, and boosting the PCE from 23.26% in the control device to 25.48% in the optimized cell with negligible hysteresis (HI = 0.0027). The LiHCOO‐engineered interface delivers remarkable stability, enabling unencapsulated devices to retain 91.3% of their initial PCE after 120 days of aging. Overall, this study demonstrates polymorph‐mediated pseudo‐halide diffusion as an effective strategy for achieving low‐loss characteristics and improved robustness in PSCs through buried interface engineering.

## 4 Experimental Section

### 4.1 Preparation of the LiHCOO Buried Interface Layer

LiHCOO solutions with concentrations of 5, 10, 15, 20, and 25 mg mL^−1^ were prepared by dissolving the corresponding masses of LiHCOO in 1 mL of formic acid, aided by the addition of a small amount of deionized water to promote complete dissolution. The resulting solutions were spin‐coated onto the SnO_2_ layer at 5000 rpm for 30 s under a nitrogen atmosphere. The films were then annealed on a hot plate at 160°C, 240°C, or 320°C for 5 min, followed by rapid cooling to room temperature. This thermal process yields LiHCOO‐H, LiHCOO‐H/M, and LiHCOO‐M polymorphs at the SnO_2_ surface prior to perovskite deposition, which serve as the initial buried interface modification states for subsequent perovskite growth.

### 4.2 Preparation of the Perovskite

The (FAPbI_3_)_0.95_(MAPbBr_3_)_0.05_ precursor solution was prepared by mixing 1.2 m (mol L^−1^) FAPbI_3_ and 1.2 m MAPbBr_3_ solutions at a volume ratio of 95:5. The precursor composition consisted of FAI (196.0 mg), PbI_2_ (551.8 mg), MABr (6.7 mg), PbBr_2_ (22.0 mg), and MACl (10 mg) dissolved in a mixed DMF:DMSO (4:1, v/v) solvent system to yield a total volume of 1 mL. The precursor was spin‐coated onto the LiHCOO‐H, LiHCOO‐H/M, and LiHCOO‐M modified buried interfaces at 2000 rpm for 5 s and subsequently at 5000 rpm for 20 s. During the final 10 s of the high‐speed step, 120 µL of chlorobenzene (CB) was dripped onto the spinning substrate as an antisolvent. The resulting perovskite films were then annealed at 110°C for 20 min inside a nitrogen‐filled glovebox.

More details on the device fabrication are provided in the Supporting Information.

## Author Contributions

C.G. contributed to conceptualization, perovskite device fabrication and characterization, data processing and analysis, and drafted and revised the manuscript. L.H. conducted PL measurements and drafted and revised the manuscript. C.L. performed XPS measurements and reviewed and revised the final manuscript. L.F. carried out XRD analysis, SEM measurements, TOF‐SIMS analysis, AFM measurements, and TRPL analysis. W.S. conceived and supervised the project. All authors discussed the experimental parameters and commented on the manuscript.

## Conflicts of Interest

The authors declare no conflicts of interest.

## Supporting information




**Supporting File**: advs75864‐sup‐0001‐SuppMat.docx.

## Data Availability

The data that support the findings of this study are available from the corresponding author upon reasonable request.
