# Supplementary material for: In Situ Pseudo‐Halide Diffusion Enables Buried Interface Regulation and Crystallinity Enhancement in Perovskite Solar Cells
Source: Adv Sci (Weinh). 2026 May 29:e75864. Online ahead of print. doi: 10.1002/advs.75864 (PMC13335929; doi:10.1002/advs.75864)
Supplement: Supplementary file 1 — Supporting File: advs75864‐sup‐0001‐SuppMat.docx. [file ADVS-9999-e75864-s001.docx]

**Supporting Information**

***In-situ* Pseudo-halide Diffusion Enables Buried Interface Regulation and Crystallinity Enhancement in Perovskite Solar Cells**

Chao Gao^a, c, 1^, Li He ^b, c, 1, *^, Changjiang Li^a^, Long Fang^d, *^, Wenzhong Shen^c, *^

^a^ College of Chemistry and Chemical Engineering, Huangshan University, Huangshan 245041, P. R. China

^b^ Key Laboratory of Functional Materials Physics and Chemistry of the Ministry of Education, Jilin Normal University, Changchun 130013, P. R. China

^c^ Institute of Solar Energy, and Key Laboratory of Artificial Structures and Quantum Control (Ministry of Education), School of Physics and Astronomy, Shanghai Jiao Tong University, Shanghai 200240, P. R. China

^d^ Renewable Energy School, Inner Mongolia University of Technology, Ordos 017000, P. R. China

^1^ These authors contributed equally to this work.

* Corresponding author, **E-mail:** jlnuhl@126.com, longfang@imut.edu.cn, wzshen@sjtu.edu.cn

**Experimental Section**

**Materials:**

Liquid reagents N,N-dimethylformamide (DMF, 99.8%, anhydrous), dimethylsulfoxide (DMSO, 99.8%, anhydrous), chlorobenzene (CB，99.8%, anhydrous), and lithium formate hydrate (LiHCOO·H_2_O) were purchased from Sigma-Aldrich. HCOOH was purchased from Aladdin. SnO_2_ aqueous colloidal dispersion (15 wt%) was purchased from Alfa Aesar. Lead diiodide (PbI_2_), and lead dibromide (PbBr_2_) were purchased from Tokyo Chemical Industry Co., Ltd. Formamidine iodide (FAI), methylammonium bromide (MABr), and methylammonium chloride (MACl) were purchased from GreatCell Solar. Spiro-OMeTAD, 4-tert-butylpyridine (tBP), and lithium bis(trifluoromethane)sulfonimide (Li-TFSI) were purchased from Advanced Election Technology Co., Ltd. All materials were used directly without further purification.

**Device Fabrication:**

Patterned ITO glass substrates (0.7 mm thick, 1.7 cm × 1.7 cm, *R*_s_ ≤ 10 Ω sq^-1^) were sequentially cleaned in ultrasonic baths of deionized water, acetone, isopropanol (IPA), and ethanol for 20 min each. After drying, the substrates were treated with ultraviolet-ozone (UVO) for 20 min. For the SnO_2_ electron transport layer, a 15 wt% SnO_2_ colloidal dispersion was diluted threefold with deionized water, filtered through a PES membrane, and spin‐coated onto the patterned ITO at 5000 rpm for 30 s. The resulting films were annealed at 160 °C for 20 min and subjected to an additional 10 min UVO treatment. Lithium formate buried interface layers were fabricated by dissolving LiHCOO in 1 mL of formic acid to obtain precursor solutions with concentrations of 5, 10, 15, 20, and 25 mg mL^-1^. A small amount of deionized water was added to facilitate complete dissolution. The solutions were spin-coated onto the SnO_2_ substrate at 5000 rpm for 30 s under a nitrogen atmosphere, followed by thermal annealing on a hot plate at 160, 240, or 320 °C for 5 min and rapid quenching to room temperature. These thermal treatments generated the hexagonal LiHCOO-H phase, a mixed hexagonal/monoclinic LiHCOO-H/M phase, and the monoclinic LiHCOO-M phase, which served as buried interface modification layers for subsequent perovskite crystallization. After the residual formic-acid vapor was completely removed in the nitrogen-filled glovebox, the perovskite (FAPbI_3_)_0.95_(MAPbBr_3_)_0.05_ absorber layer was deposited in situ using a standard one-step antisolvent method. The perovskite precursor solution was prepared by mixing 1.2 M FAPbI_3_ and 1.2 M MAPbBr_3_ solutions at a 95:5 volume ratio. The precursor consisted of FAI (196.0 mg), PbI_2_ (551.8 mg), MABr (6.7 mg), PbBr_2_ (22.0 mg), and MACl (10 mg) dissolved in a DMF:DMSO (4:1, v/v) solvent mixture to yield a total volume of 1 mL. The precursor was spin-coated onto the LiHCOO-H, LiHCOO-H/M, and LiHCOO-M modified interfaces at 2000 rpm for 5 s and then 5000 rpm for 20 s, during which 120 μL of chlorobenzene (CB) was dripped onto the substrate in the final 10 s as an antisolvent. The perovskite films were annealed at 110 °C for 20 min inside the nitrogen glovebox. Subsequently, the spiro-OMeTAD hole transport layer was deposited by spin-coating a solution containing 72.3 mg spiro-OMeTAD, 28.8 μL tBP, 17.5 μL Li-TFSI stock solution (520 mg Li-TFSI in 1 mL acetonitrile), and 1 mL CB at 4000 rpm for 30 s. Finally, Ag top electrodes (110 nm) were thermally evaporated through a shadow mask (active area 0.052 cm^2^) under a vacuum of < 3×10^-4^ Pa.

**Characterizations:**

The surface morphologies of the perovskite films were measured using field-emission SEM (Zeiss Ultra Plus). The AFM and KPFM results were tested using the MFP-3D equipment. UPS and XPS measurements were performed in an imaging photoelectron spectrometer (Thermo Escalab 250) in an ultrahigh vacuum chamber of < 4 × 10^-8^ Pa. UPS was equipped with an ultraviolet light source (He I = 21.22 eV) with an energy resolution of 50 meV. XPS used a monochromatic Al K*α* (1486.6 eV) excitation source. TOF-SIMS was performed by ION-TOF GmbH/ TOF-SIMS 5-100 system with an analysis area of 100 × 100 μm^2^. The crystal structures of perovskite films with and without lithium formate buried interface modification were measured by XRD (Bruker, D8 ADVANCE Da Vinci) using a Cu K*α* radiation source (*λ* = 0.15406 nm). Hyperspectral PL mapping spectra were measured using the TESCAN/ RISE-MAGNA instrument. The steady-state PL of the samples was measured by a Raman spectrometer (Horiba Jobin Yvon, LABRAM HR800) with an excitation at 514.5 nm using an argon-ion laser, and the TRPL was detected by a fluorescence spectrometer (Edinburgh Instruments, FLS1000). The UV-vis absorption spectra were obtained with a UV-vis-NIR spectrophotometer (PerkinElmer, Lamda 950). EQE measurements were performed on a quantum efficiency/IPCE system (PV Measurement, QEX10) in the 300-850 nm wavelength range. The photocurrent density-voltage (*J-V*) characteristics of the PSCs were measured using a programmable Keithley 2400 source meter under illumination from a 300 W class AAA solar simulator (100 mW cm^-2^, Newport Oriel, Model 94043A). The water contact angle was measured by using the optical contact angle measuring instrument (Kruss, DSA 100).

**First-principles calculations**

Density functional theory (DFT) calculations were performed using the Vienna ab initio simulation package (VASP).^[1,2]^ The projector augmented-wave (PAW) method and the Perdew-Burke-Ernzerhof (PBE) functional within the generalized gradient approximation (GGA) were employed to describe the electron-ion and exchange-correlation interactions.^[3,4]^ A bulk FAPbI_3_ supercell was constructed from the CIF structure obtained from the ICSD database,^[5]^ and the substitution of iodide with formate anions was modeled under fully periodic boundary conditions. Geometry optimization was carried out with relaxation of both atomic coordinates and lattice parameters until the forces on all atoms were below 0.02 eV Å^-1^ and electronic energy convergence criterion reached 10^-6^ eV. A plane-wave energy cutoff of 500 eV was used in the calculations. Long-range van der Waals interactions were accounted for using the Grimme’s DFT-D3 dispersion correction.^[4,6,7]^

**Note S1.**

The spatial distribution of optical absorption (Abs.) within the model can be obtained from the electric field *E* according to:

$$\begin{aligned} Abs.=-0.5\omega|E|^{2}\mathrm{imag} \left( N \right)\#(S1) \end{aligned}$$

where *ω* is the angular frequency, *N* is the complex refractive index.

The average optical path length *P̅* can then be calculated using Equation (S2) reported by Campbell *et al.*^[8]^:

$$\begin{aligned} \bar{P}=\frac{2W\left( 1+R \right)}{\left[ 1-R\left( 1-f \right) \right]}\#(S2) \end{aligned}$$

where *W* is the thickness of the perovskite layer, *R* is the reflectance at the bottom surface of the perovskite, and *f* represents the fraction of light escaping from the top surface.

**Note S2.**

We employed a bi-exponential function to fit the TRPL decay curves, which consists of a fast decay component (*τ*_1_) and a slow decay component (*τ*_2_). The average carrier lifetime (*τ*_avg_) was calculated using Equation (S3):

$$\begin{aligned} \text{τ}_{\text{avg}}\text{ = }\frac{\left( \text{A}_{\text{1}}\text{τ}_{\text{1}}^{\text{2}}\text{+}\text{A}_{\text{2}}\text{τ}_{\text{2}}^{\text{2}} \right)}{\left( \text{A}_{\text{1}}\text{τ}_{\text{1}}\text{+}\text{A}_{\text{2}}\text{τ}_{\text{2}} \right)}\#\left（ S3 \right） \end{aligned}$$

where *A*_1_ and *A*_2_ are the fitting amplitudes.

**Note S3.**

The built-in potential (*V*_bi_) and carrier density (*N_D_*) at equilibrium can be extracted from the Mott-Schottky plot using the depletion-region expression:

$$\begin{aligned} \left( \frac{C}{A} \right)^{2}=\frac{q\varepsilon_{r}\varepsilon_{0}N_{D}}{2\left( V_{bi}-V \right)}\#\left( S4 \right) \end{aligned}$$

where *C* is the depletion capacitance, *A* is the effective device area, *V* is the applied bias, *q* is the elementary charge, *ɛ_r_* is the relative permittivity of the perovskite, and *ɛ*_0_ is the vacuum permittivity.

**Table S1.** Published high-performance regular n-i-p PSCs involving buried interface modification.

| Additive | Spotlight | *J*_SC_  (mA/cm^2^) | FF  (%) | *V*_OC_  (V) | PCE  (%) | Bandgap | Ref. |
| --- | --- | --- | --- | --- | --- | --- | --- |
| Sodium gluconate (SG) | Bridging additive suppressing buried-interface pinholes and enhancing contact quality. | 25.76 | 82.38 | 1.194 | **25.34** | 1.55 | [9] |
| FBI-PyAI | Soft arch-bridge molecule enabling stress buffering, defect passivation, and oriented crystallization. | 26.32 | 80.50 | 1.18 | **25.01** | 1.56 | [10] |
| SDBA (4,4′-sulfonyldibenzoic acid) | Active passivator enabling vacancy healing, improved SnO_2_ nucleation, and optimized ETL band alignment. | 26.22 | 85.49 | 1.16 | **25.94** | 1.55 | [11] |
| MeOBTBT-POEt | Supramolecular template enabling (100)-oriented growth, defect passivation, and minimized *V*_OC_ deficit. | 25.10 | 82.28 | 1.228 | **25.34** | 1.53 | [12] |
| Potassium 4-methoxysalicylate (MSAK) | MSAK provides strong COO^-^-based bridging to passivate SnO_2_/PVK defects and improve crystallization. | 25.58 | 83.94 | 1.186 | **25.47** | 1.55 | [13] |
| 4-vinylbenzoic acid (VA) | Polymerized molecular zipper strengthens adhesion and passivates buried-interface defects. | 25.55 | 80.71 | 1.186 | **24.47** | 1.55 | [14] |
| CS-103 | Dual-anchoring dipolar bridge for synchronous defect passivation and energy-level alignment. | 25.64 | 83.23 | 1.161 | **24.77** | 1.55 | [15] |
| NaTSA (sodium trifluoromethanesulfonate) | Dual-anchoring sulfonate enables strongest defect passivation and optimal energy alignment. | 25.60 | 83.2 | 1.186 | **25.60** | 1.57 | [16] |
| DL-Ethionine (DLEO) | Insoluble molecular bridge enabling stable defect passivation and improved crystallization. | 26.05 | 83.95 | 1.146 | **25.08** | 1.55 | [17] |
| EATsO | Self-assembled sulfonate enabling dual-side passivation and 2D-seed guided crystallization. | 25.91 | 82.73 | 1.19 | **25.51** | 1.535 | [18] |
| C3F7-MA | Push-pull π-molecule enabling dual-defect passivation and reduced interfacial energy barrier. | 23.88 | 82 | 1.241 | **24.16** | 1.60 | [19] |
| F-ISS | FA-assisted in-situ coordination enabling defect healing, stress relief, and stronger buried-interface adhesion. | 25.69 | 83.11 | 1.20 | **25.61** | 1.55 | [20] |
| Ectoine (Ec) | Dual-anchoring molecular bridge flattens grain-boundary grooves and releases residual stress. | 25.43 | 81.85 | 1.186 | **24.68** | 1.56 | [21] |
| 4,4′-Bipyridine | Dual-side defect passivation and reduced surface energy enabling uniform nucleation and improved buried-interface crystallinity. | 25.38 | 84.2 | 1.205 | **25.75** | 1.53 | [22] |
| HBPDC (2,2′-bipyridyl-4,4′-dicarboxylic acid) | Dual-anchoring molecule enabling double-side defect passivation and improved energy alignment. | 25.53 | 83.6 | 1.191 | **25.41** | 1.57 | [23] |
| _L_-Citrulline (CIT) | Dual-side chemical bridge enabling strong adhesion, deep defect passivation, and enlarged perovskite grains. | 26.36 | 81.97 | 1.201 | **25.95** | 1.55 | [24] |
| CNCB (2-cyano-N,N,N-trimethylammonium bromide) | High-polarity molecular bridge enabling crystallization control, dual-defect passivation, and dipole-induced energy alignment. | 25.91 | 84.25 | 1.21 | **26.47** | 1.552 | [25] |
| IMA + BTFBT | S_N_2 chemical bonding enabling strong buried adhesion and (100)-oriented crystallization. | 14.576 | 78.59 | 1.322 | **15.14** | 1.90 | [26] |
| Ph-Tz (1,4-di(thiazol-2-yl)benzene) | Dual-site dipole molecule enabling strong defect suppression, improved energy alignment, and enhanced carrier extraction. | 25.51 | 86.41 | 1.178 | **25.85** | 1.55 | [27] |
| Bisphenol S (BPS) | Crosslinked SnO_2_ network enabling oxygen-defect mitigation and (100)-oriented perovskite growth. | 25.04 | 83.76 | 1.18 | **24.87** | 1.56 | [28] |
| TFSK (potassium trifluoromethanesulfonate) | Multifunctional sulfonate enabling defect passivation, porous PbI_2_ formation, improved crystallization, and minimized voltage loss. | 26.06 | 83.33 | 1.19 | **25.82** | 1.53 | [29] |
| CsTFA (cesium trifluoroacetate) | Bidirectional coordination enabling vertical crystal growth, strain release, and low-defect buried interface. | 26.00 | 83.81 | 1.175 | **25.60** | 1.52 | [30] |
| PbCl_2_ (via in-situ generated PMC: Pb(CH_3_NH_2_)_2_Cl_2_ interphase) | Metastable interphase providing pre-compressive strain to suppress defects and enable strain-free perovskite growth. | 26.14 | 83.6 | 1.182 | **25.83** | 1.55 | [31] |
| CyP (acesulfame potassium) | Cyclic passivator enabling dual-side defect passivation, enhanced carrier extraction, and record CsPbI_3_ QD device efficiency. | 18.09 | 77.3 | 1.251 | **17.5** | 1.80 | [32] |
| PC (phenylhydrazinium chloride) | PbI_2_‐binding modifier enabling defect passivation, slowed crystallization, strain relief, and high-quality buried interface. | 25.43 | 85.56 | 1.186 | **25.80** | 1.57 | [33] |
| TBA (3-thiopheneboronic acid) and PBA (4-pyridineboronic acid) | Electron-withdrawing SAM (PBA) suppresses radiative recombination, while electron-donating SAM (TBA) aggravates it despite defect passivation. | 25.08 | 82.90 | 1.11 | **23.08** | 1.52 | [34] |
| D-Methionine (D.M) | Dual-functional molecular bridging for defect passivation and energy alignment. | 25.96 | 83 | 1.17 | **25.39** | 1.55 | [35] |
| NaF (best-performing) and LiF | Alkali fluorides enhance SnO_2_ wettability and passivate buried-interface defects, enabling larger grains and reduced recombination. | 24.62 | 81.05 | 1.162 | **23.19** | 1.59 | [36] |
| LiAc (Lithium acetate) | LiAc enables dual-function defect passivation and n-type doping for improved buried-interface quality. | 25.54 | 82.91 | 1.203 | **25.48** | 1.55 | [37] |
| MESK (2-(N-morpholino)ethanesulfonic acid potassium salt) | Bidirectional sulfonate bridge enabling dual-side defect passivation, improved SnO_2_ transport, and out-of-plane perovskite orientation. | 24.77 | 84.08 | 1.18 | **24.67** | 1.53 | [38] |
| IT-4F | Energy-level tuning + stress relaxation enabling enhanced charge extraction and reduced nonradiative loss. | 24.87 | 81.56 | 1.17 | **23.73** | 1.55 | [39] |
| CL-NH (organic-inorganic complex) | Multifunctional complex enabling oxygen-vacancy removal and crystallization control for dual-side defect passivation. | 25.22 | 79.12 | 1.187 | **23.69** | 1.56 | [40] |
| HPDA (hyperbranched polymer with dopamine end groups) | 3D dopamine-anchored polymer crosslinks grain boundaries and interfaces, releasing strain and greatly enhancing mechanical durability. | 25.82 | 85.0 | 1.178 | **25.92** | 1.56 | [41] |
| DTPA (diethylenetriaminepentaacetic acid) + Zol (zolephonic acid) | Fully chemical crosslinked layer preventing modifier detachment, enabling strong charge extraction and exceptional static/dynamic stability. | 25.59 | 84.51 | 1.18 | **25.52** | 1.54 | [42] |
| Heparin sodium (HS) | Multifunctional polymer bridge enabling strong chemical bonding, dual-side defect passivation, and stress relief. | 26.30 | 85.82 | 1.179 | **26.61** | 1.52 | [43] |
| DHIII (2,3-dihydroisoindole hydroiodide) | Spontaneously formed buried 2D perovskite enables uniform crystallization, strain relaxation, and strong defect passivation. | 26.20 | 83.96 | 1.196 | **26.31** | 1.53 | [44] |
| DHHB (diethylamino hydroxybenzoyl hexyl benzoate) | Multifunctional molecule enabling UV shielding + strain regulation + defect passivation for highly stable buried interface. | 26.39 | 84.37 | 1.189 | **26.47** | 1.55 | [45] |
| 4-Chloro-3-sulfamoylbenzoic acid (CSBA) | Oriented molecular bridge enabling dual-side passivation, improved energy alignment, and strain release. | 26.21 | 82.97 | 1.164 | **25.32** | 1.52 | [46] |
| HCOONH_4_ (ammonium formate) | Pre-buried volatile additive enabling cross-layer defect reduction, strain release, and robust flexible-device durability. | 23.44 | 82.89 | 1.151 | **22.37** | 1.54 | [47] |
| *β*A (*β*-guanidinopropionic acid) | Residue-free SnO_2_ plus molecular bridge enabling strain-free crystallization and fast electron extraction. | 25.81 | 84.04 | 1.19 | **25.74** | 1.55 | [48] |
| / | Lattice-matched SrSnO_3_ ETL enables ordered epitaxial nucleation, eliminating buried-interface stress and defects. | 25.72 | 83.67 | 1.17 | **25.17** | 1.54 | [49] |
| CsCl:Eu^3+^ quantum dots | Eu^3+^-doped CsCl QDs retain the inherent bandgap while compensating lattice strain and suppressing nonradiative loss. | 26.10 | 80.39 | 1.15 | **24.13** | 1.51 | [50] |
| Allicin | Oily allicin passivates buried defects and wraps grains to suppress ion migration. | 25.72 | 84.03 | 1.16 | **25.07** | 1.538 | [51] |
| Lithium formate | Monoclinic-phase-enabled pseudo-halide diffusion for improved crystallization, defect passivation, and device performance. | 25.44 | 82.57 | 1.213 | **25.48** | 1.55 | **This work** |


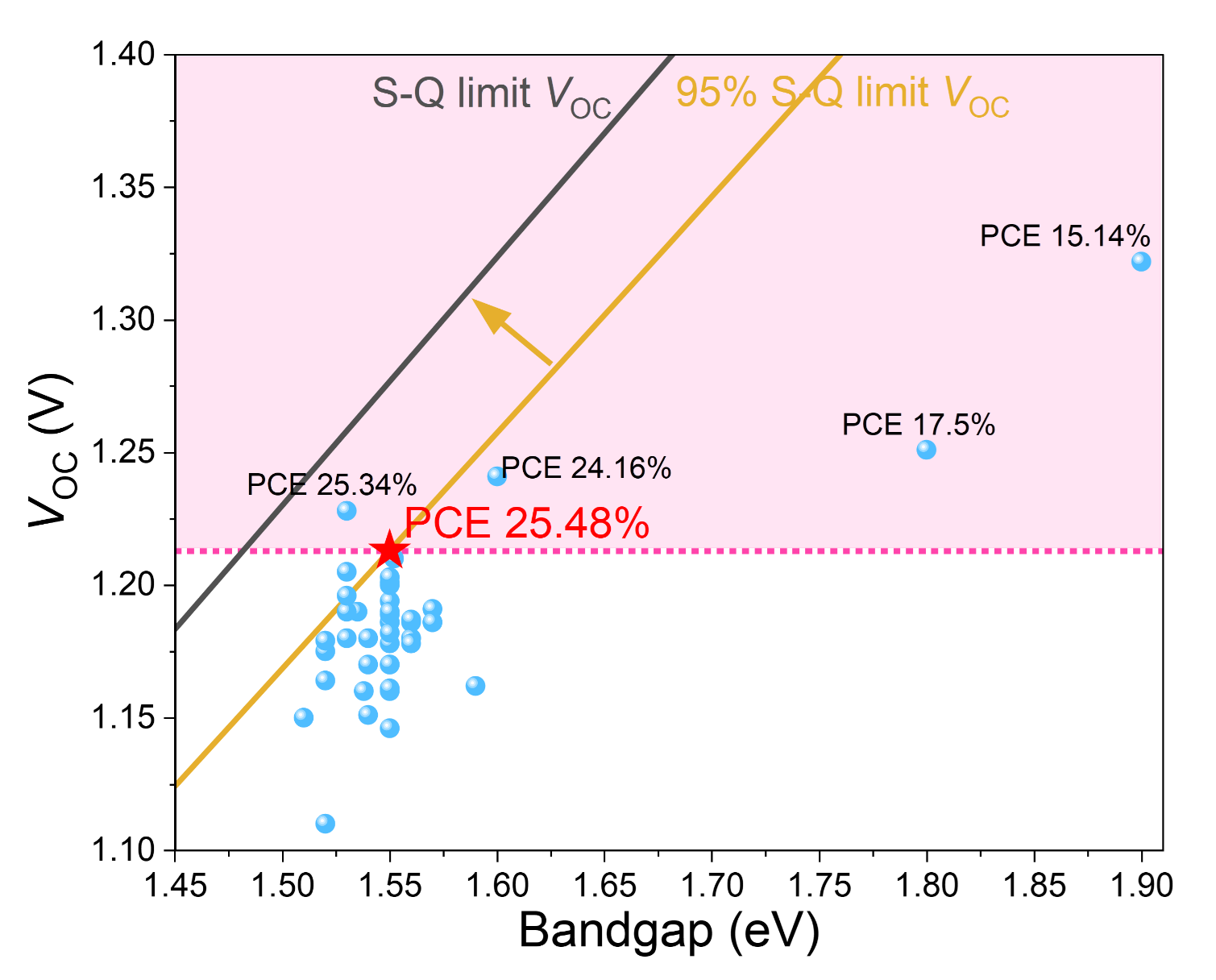


**Figure S1.** Relationship between *V*_OC_ and bandgap for regular n-i-p PSCs reported in the literature on buried interface modification. Only PCEs displaying a *V*_OC_ greater than 1.213 V (this work) are shown in the figure.


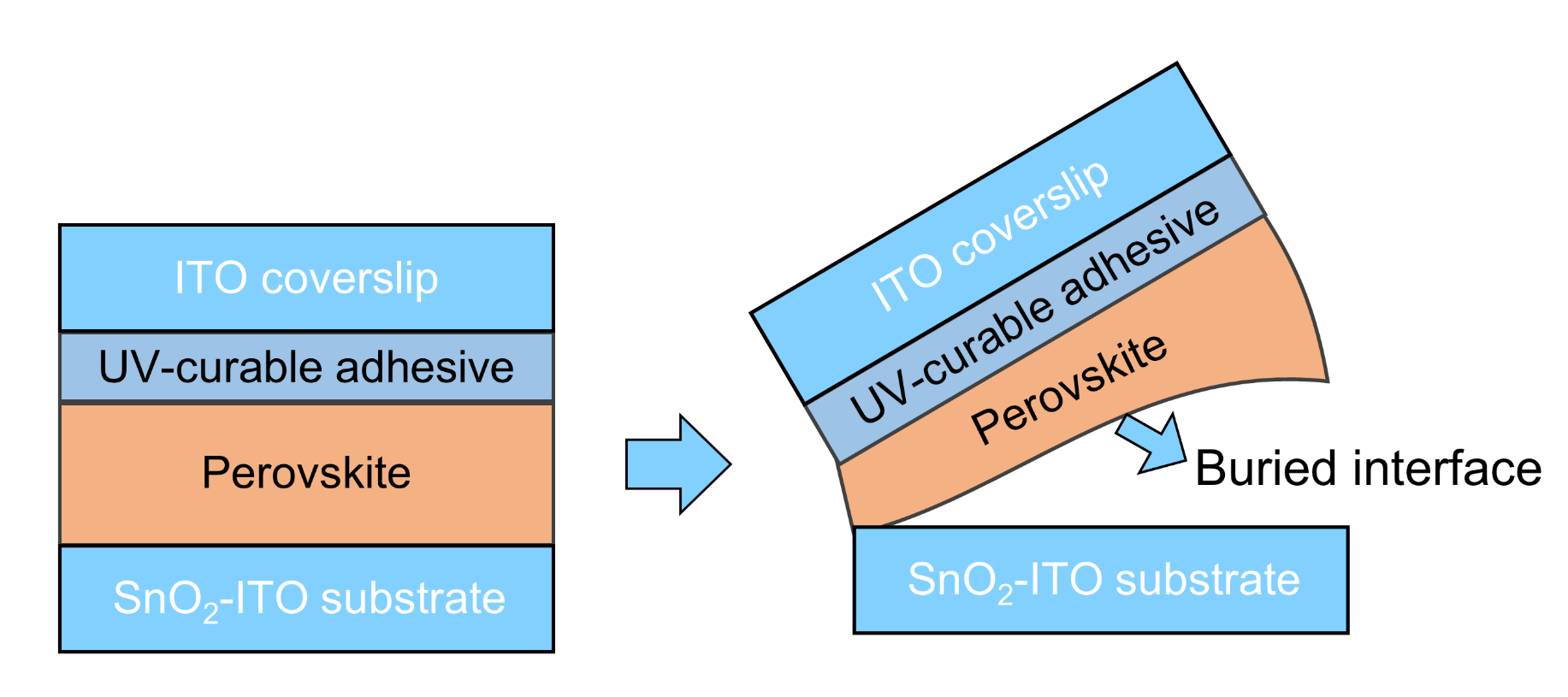


**Figure S2.** Schematic illustration of the preparation process used to expose and obtain the perovskite buried interface for direct SEM characterization.


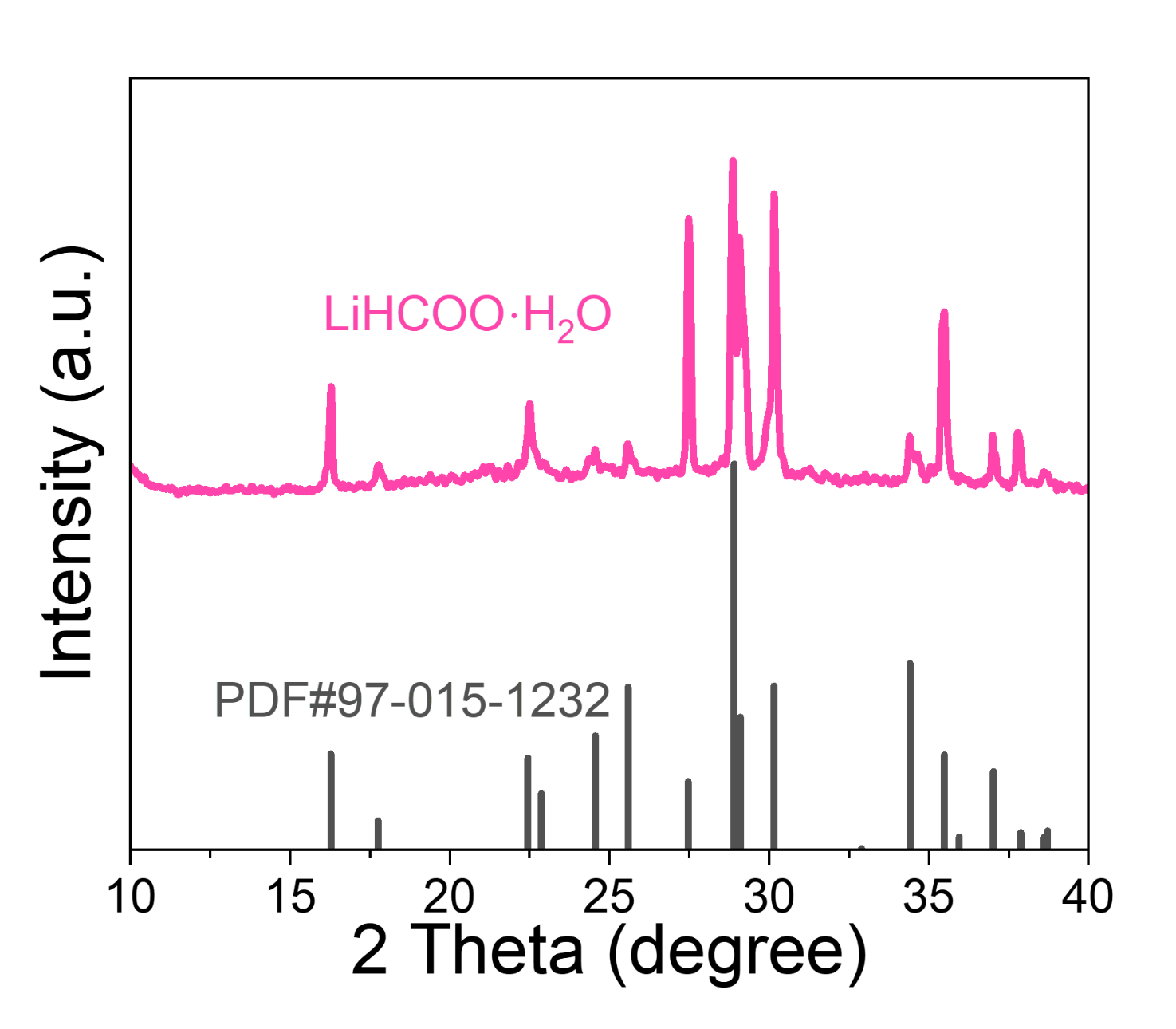


**Figure S3.** Standard XRD pattern of hydrated LiHCOO·H_2_O and the XRD pattern measured at room temperature.


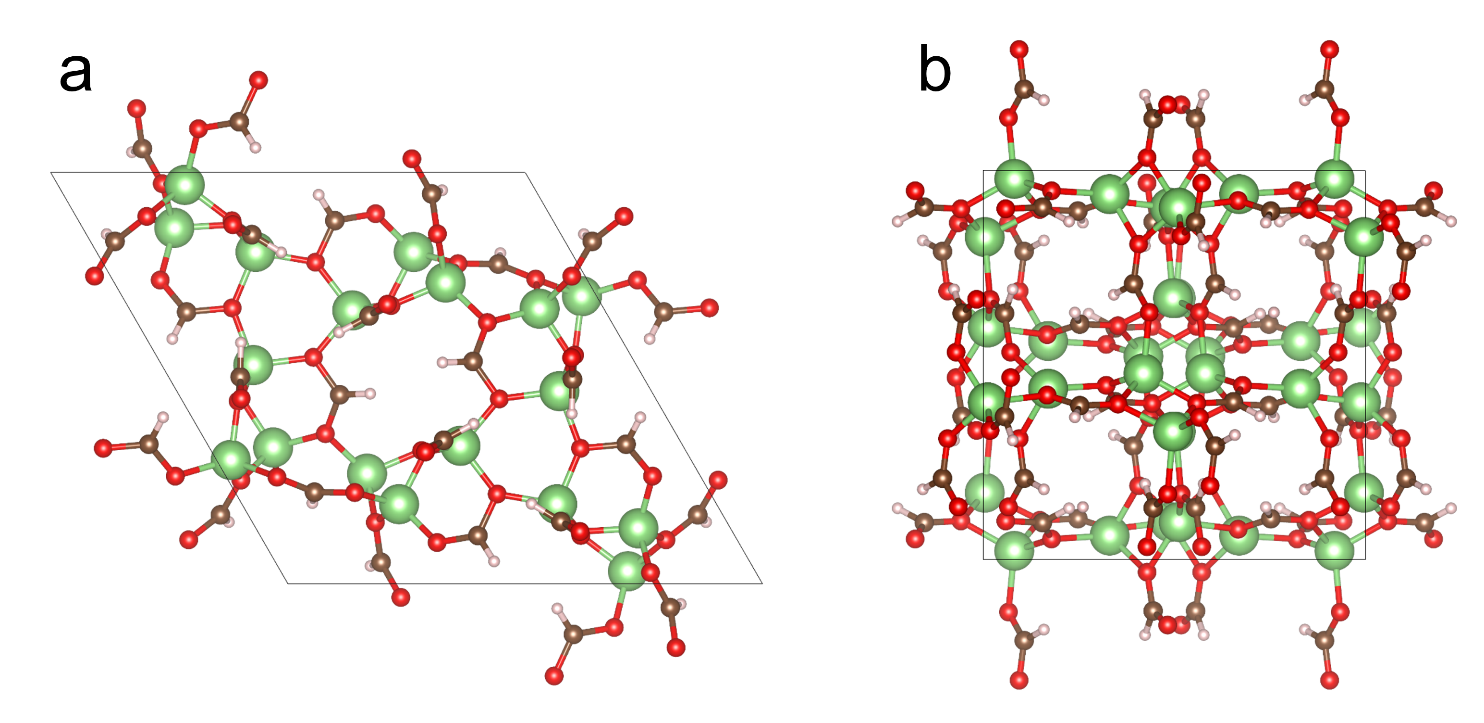


**Figure S4.** Lattice configuration diagrams of (a) LiHCOO-H and (b) LiHCOO-M obtained from the ICSD.^[5]^ The hexagonal phase of lithium formate has a density of 1.454 g cm^-3^, whereas the monoclinic phase has a density of 1.431 g cm^-3^.^[52,53]^


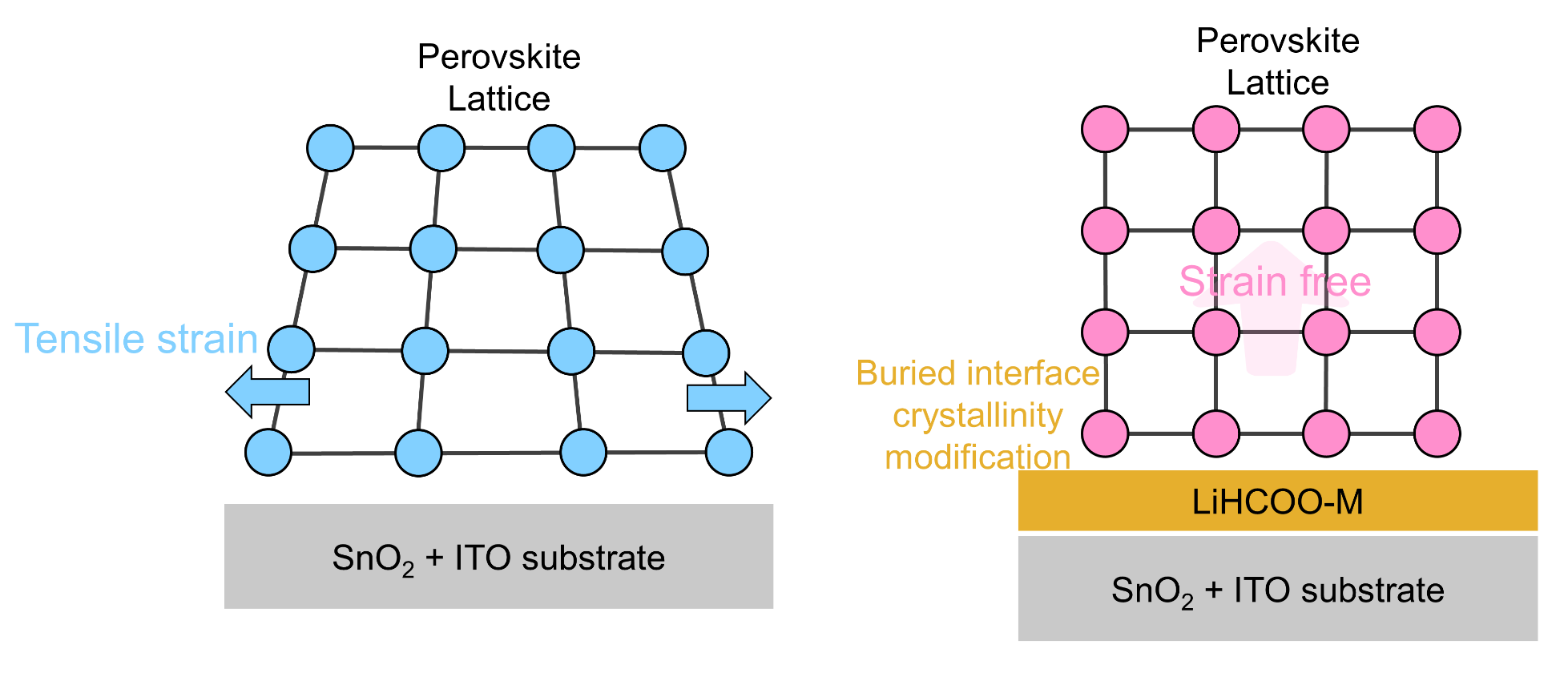


**Figure S5.** Schematic illustration of tensile strain at the buried interface in the control sample and the strain-free interface in the target sample enabled by LiHCOO-M modification.


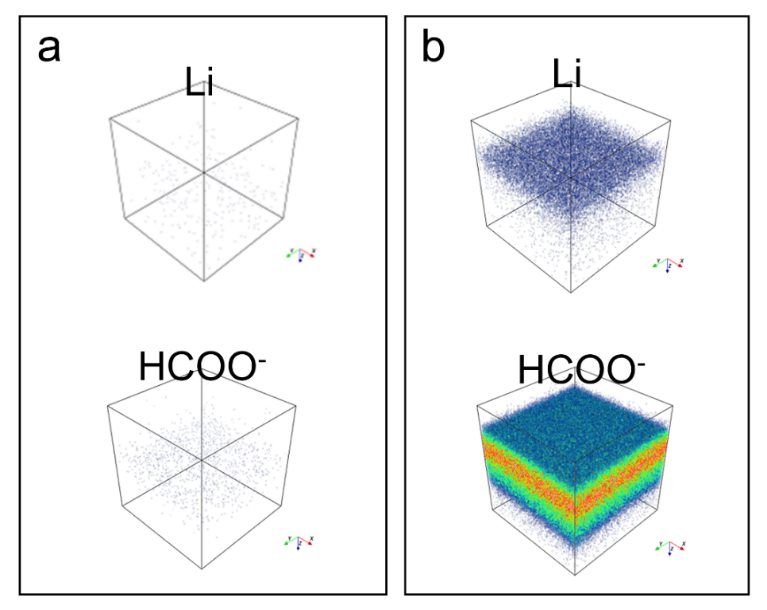


**Figure S6.** Three-dimensional TOF-SIMS spatial distribution of lithium formate for (a) the control, and (b) the target samples.


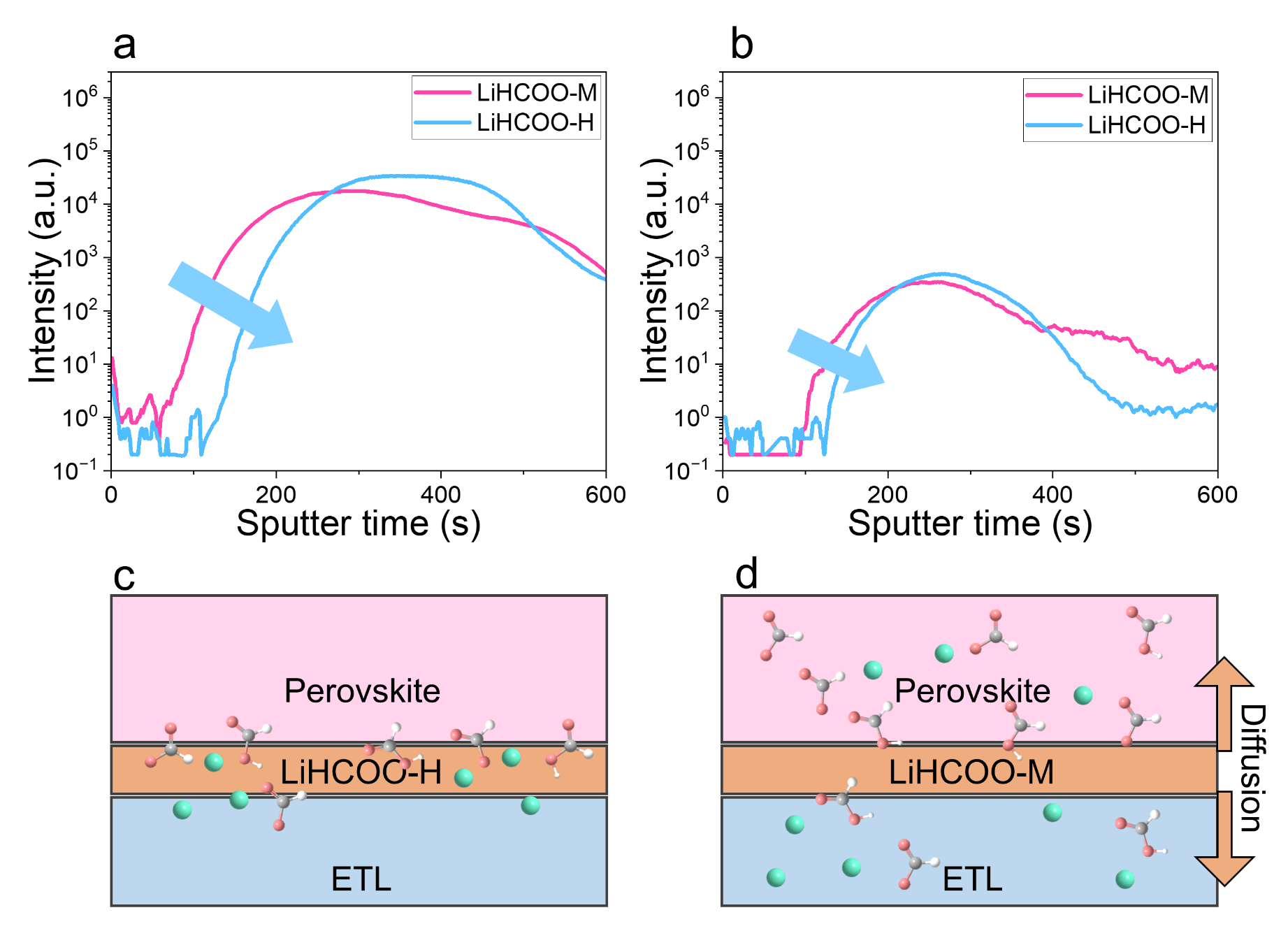


**Figure S7.** TOF-SIMS depth profiles of LiHCOO-H and LiHCOO-M samples showing (a) HCOO^-^ and (b) Li^+^. Schematic illustrations of ion diffusion at the SnO_2_/perovskite buried interface based on the TOF-SIMS results: diffusion of HCOO^-^ and Li^+^ ions in (c) the LiHCOO-H, and (d) the LiHCOO-M samples.


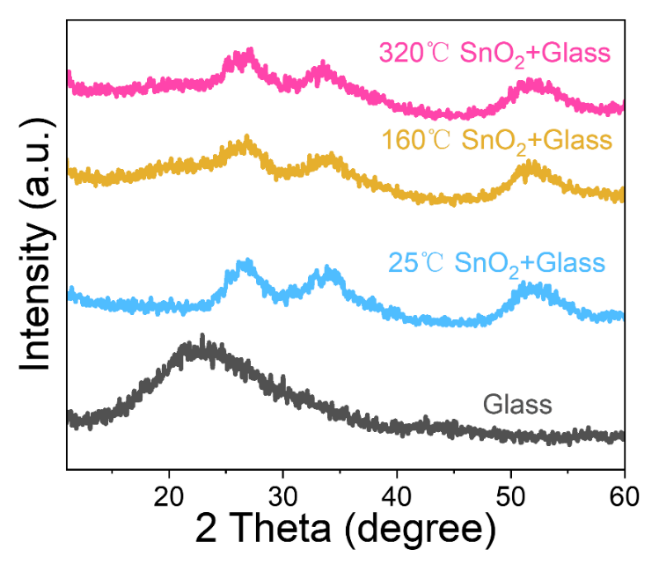


**Figure S8.** XRD patterns of SnO_2_ and glass substrates after annealing at different temperatures.


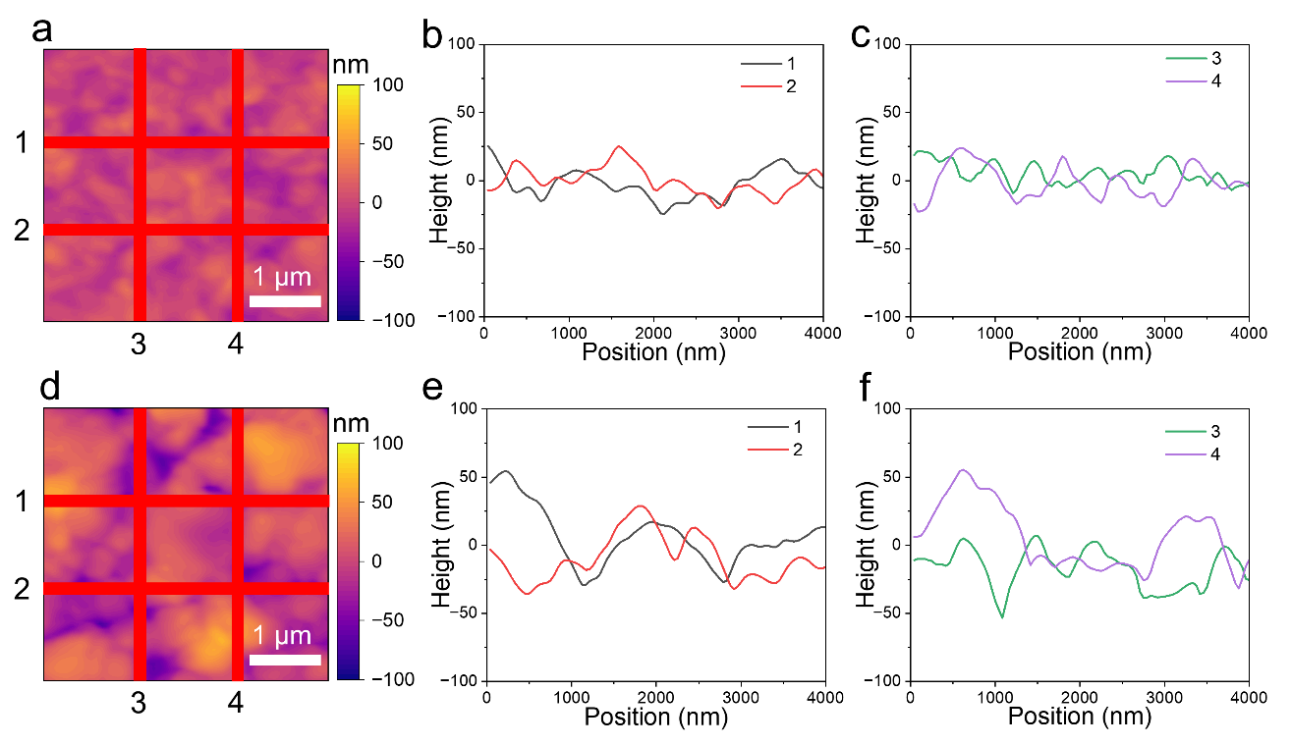


**Figure S9.** RMS roughness derived from AFM line profiles for (a-c) the control, and (d-f) the target samples. In panel (a), lines 1-2 correspond to panel (b), and lines 3-4 correspond to panel (c). Panels (d-f) follow the same correspondence.


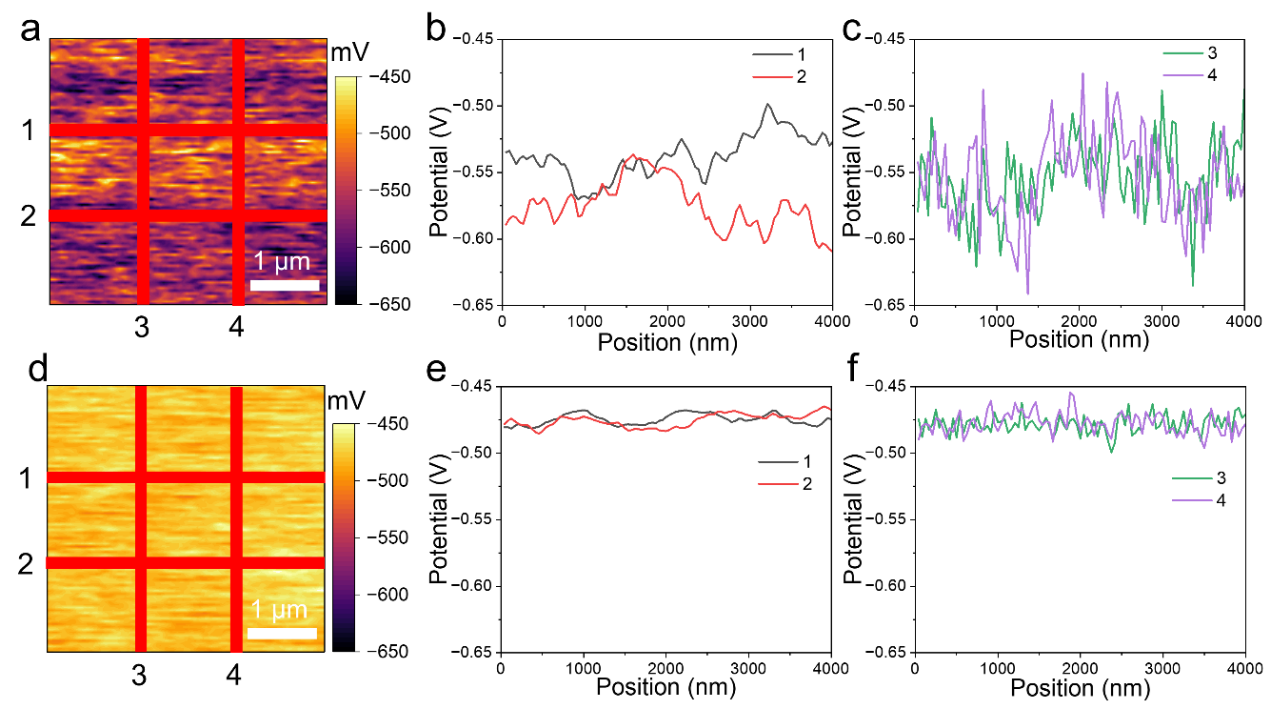


**Figure S10.** KPFM surface potential line profiles for (a-c) the control, and (d-f) the target samples. In panel (a), lines 1-2 correspond to panel (b), and lines 3-4 correspond to panel (c). Panels (d-f) follow the same correspondence.


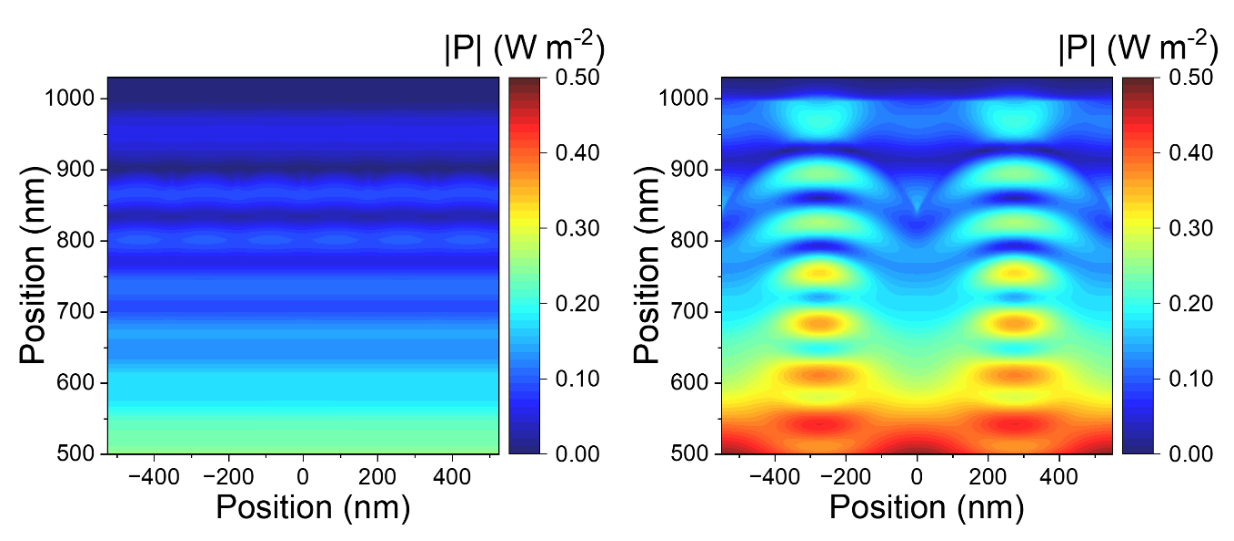


**Figure S11.** Simulated Poynting vector distributions obtained by the FDTD method for the control (left) and target (right) samples.


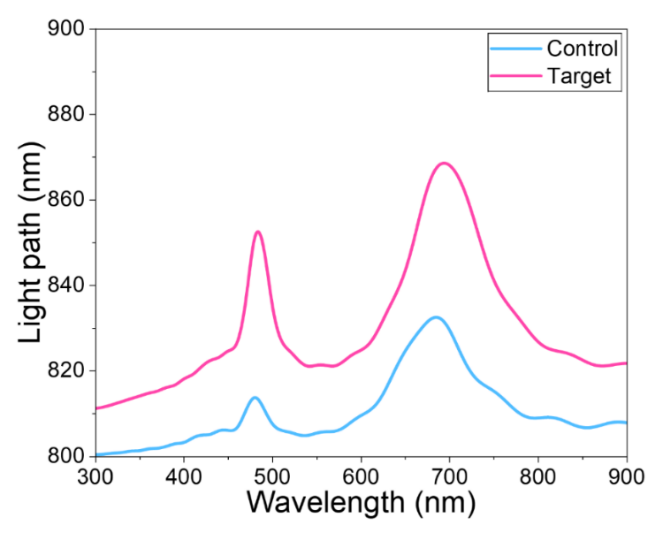


**Figure S12.** Light path lengths of the control and target samples obtained from FDTD simulations.


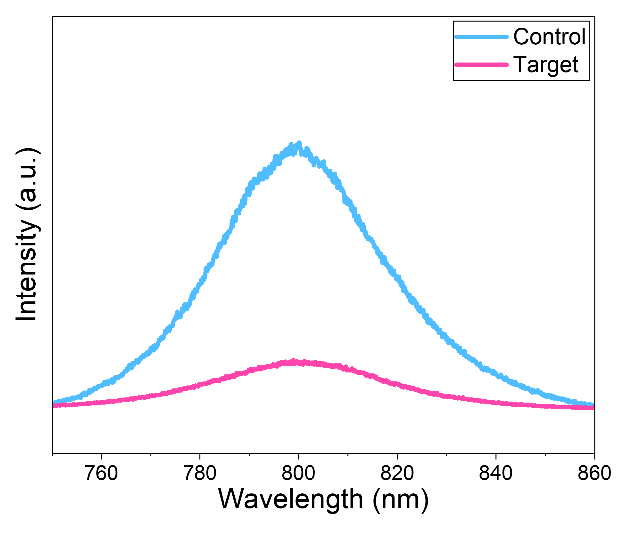


**Figure S13.** Steady-state PL spectra of the control and target PSCs.

**Table S2.** Carrier lifetimes obtained from TRPL measurements using a bi-exponential fitting model.

| **Perovskite** | ***A*_1_** | ***τ*_1_ (ns)** | ***A*_2_** | ***τ*_2_ (ns)** | ***τ*_avg_ (ns)** |
| --- | --- | --- | --- | --- | --- |
| Control | 26.39% | 42.60 | 71.41% | 257.24 | 244.86 |
| Target | 15.99% | 4.49 | 83.48% | 63.07 | 62.28 |

**Table S3.** Energy band structures of the perovskite layers in the control and target samples.

| **Conditions** | ***E_cut-off_***  **(eV)** | ***E_edge-on_***  **(eV)** | **Work function**  **(eV)** | **VBM**  **(eV)** | **CBM**  **(eV)** |
| --- | --- | --- | --- | --- | --- |
| Control | 16.97 | 1.24 | -4.25 | -5.49 | -3.94 |
| Target-top | 16.96 | 1.25 | -4.26 | -5.51 | -3.96 |
| Target-bottom | 16.90 | 1.31 | -4.32 | -5.63 | -4.08 |


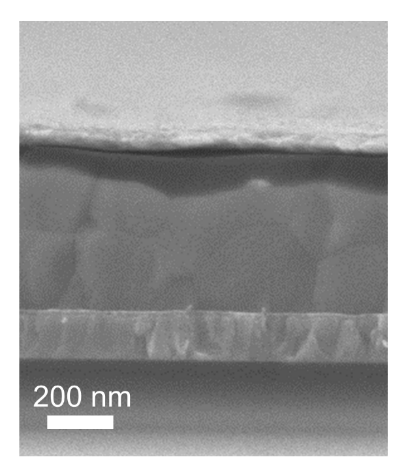


**Figure S14.** Cross-sectional SEM image of the control sample.


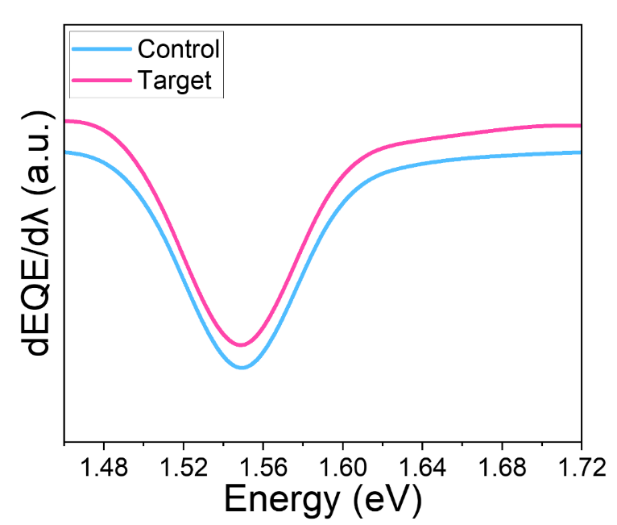


**Figure S15.** First-derivative EQE spectra of the control and target PSCs used to determine the bandgap of the devices.


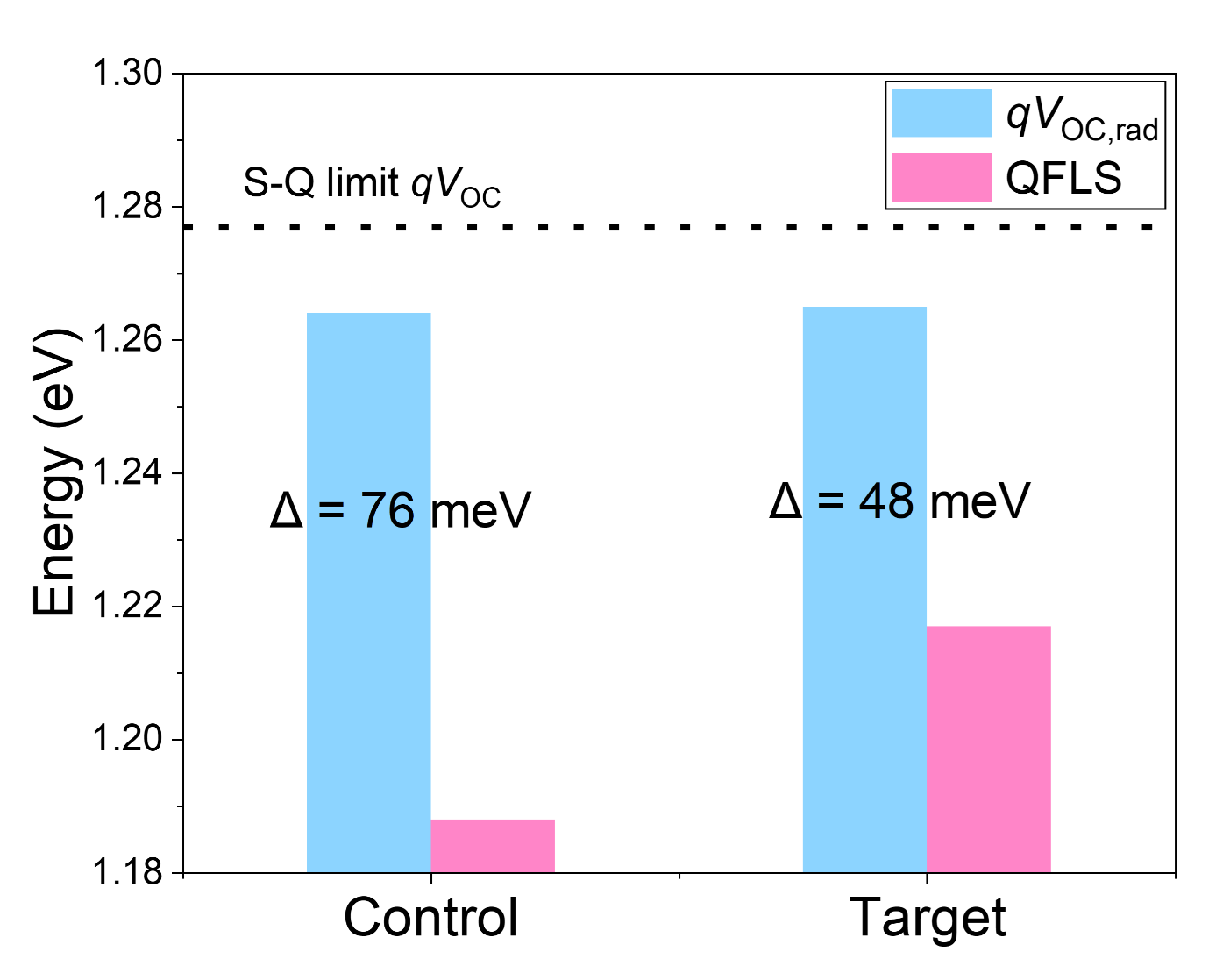


**Figure S16.** Analysis of *V*_OC_ loss for the control and target samples.


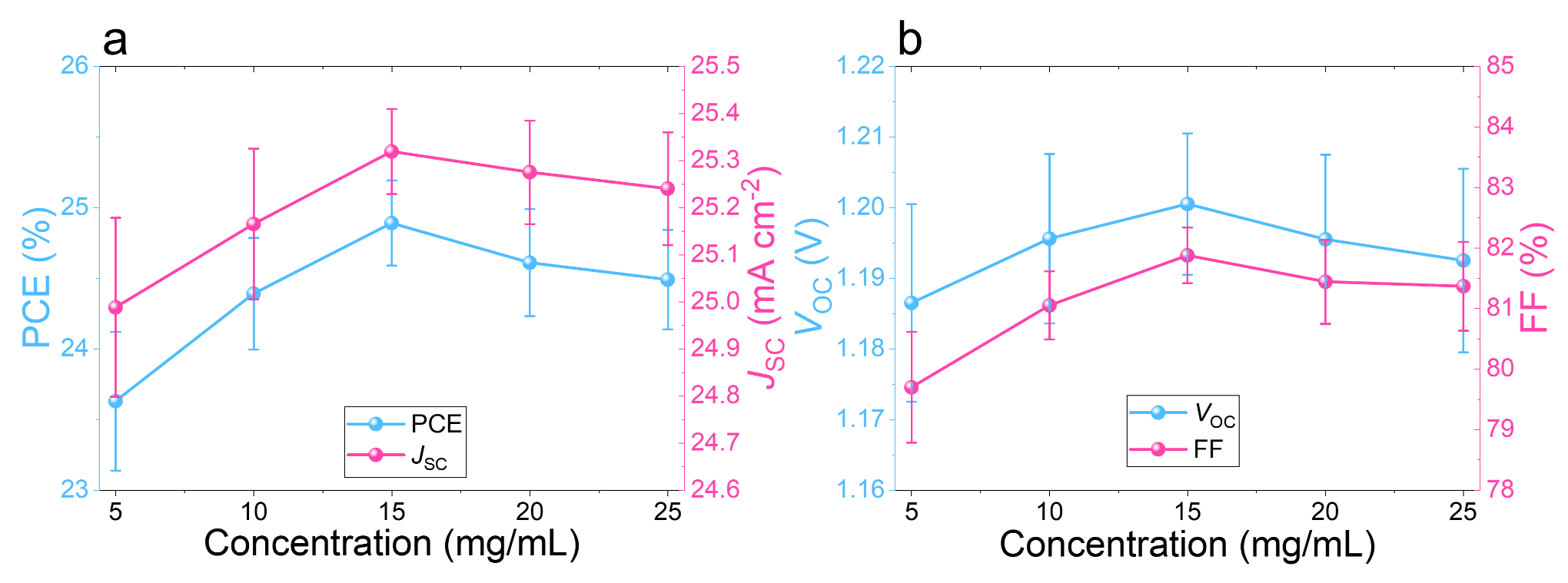


**Figure S17.** Influence of buried interfaces prepared with different lithium formate concentrations on PSC performance: (a) PCE and *J*_SC_, and (b) *V*_OC_ and FF.


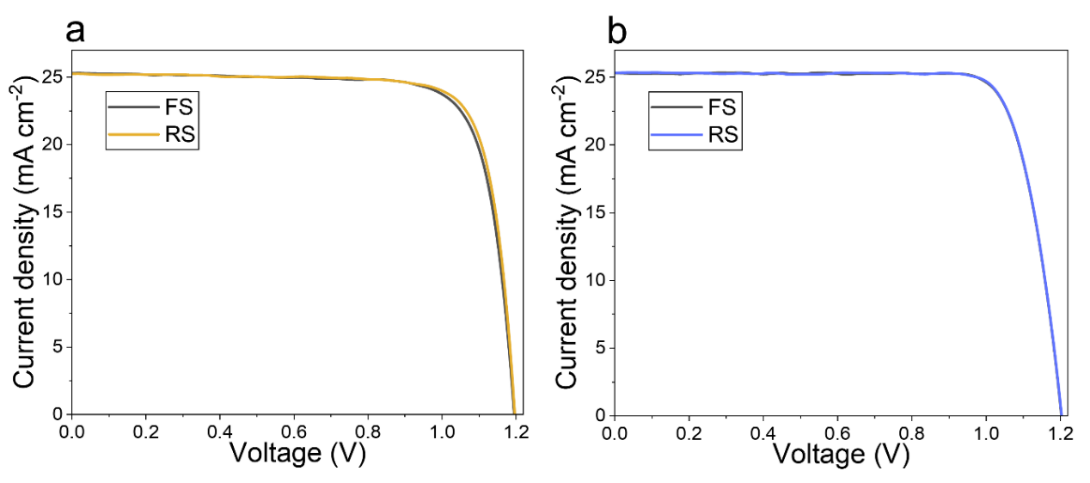


**Figure S18.** Forward and reverse *J-V* characteristics of (a) LiHCOO-H, and (b) LiHCOO-H/M devices.

**Table S4.** PV parameters of control, LiHCOO-H, LiHCOO-H/M and target cells.

| **Perovskite** | **Scan** | ***V*_OC_ (V)** | ***J*_SC_ (mA cm^-2^)** | **FF (%)** | **PCE (%)** |
| --- | --- | --- | --- | --- | --- |
| Control | RS | 1.178 | 24.94 | 79.17 | 23.26 |
|  | FS | 1.181 | 24.88 | 74.97 | 22.03 |
| LiHCOO-H | RS | 1.197 | 25.24 | 80.37 | 24.28 |
|  | FS | 1.195 | 25.28 | 78.98 | 23.86 |
| LiHCOO-H/M | RS | 1.203 | 25.32 | 81.32 | 24.77 |
|  | FS | 1.203 | 25.31 | 81.19 | 24.72 |
| LiHCOO-M  (Target) | RS | 1.213 | 25.44 | 82.57 | 25.48 |
|  | FS | 1.209 | 25.48 | 82.53 | 25.41 |


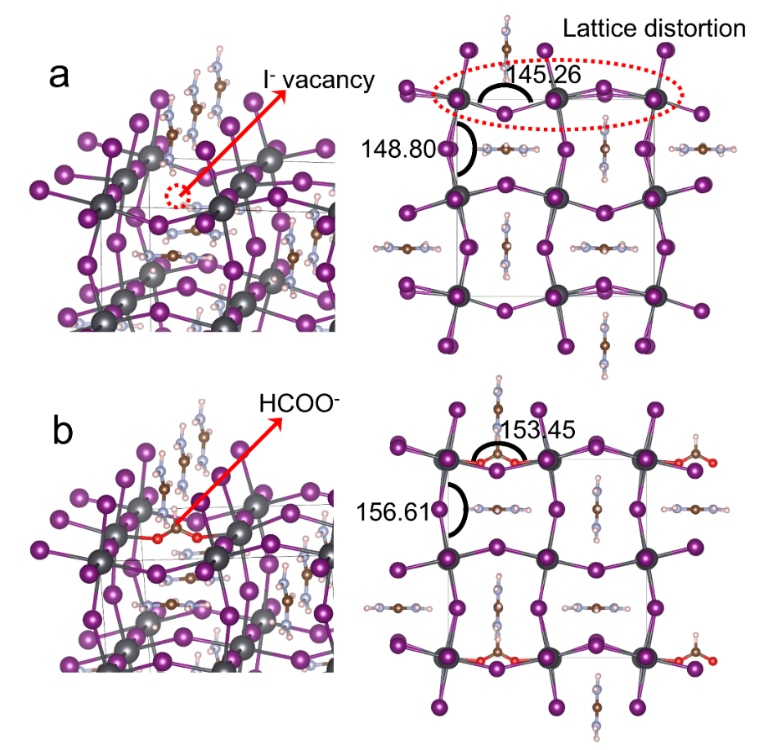


**Figure S19.** DFT-simulated results: (a) Iodine vacancies and the associated Pb-I-Pb bond angle distortion in the perovskite lattice; (b) HCOO^-^ incorporation compensating iodine vacancies and yielding the optimized Pb-I-Pb bond angle.


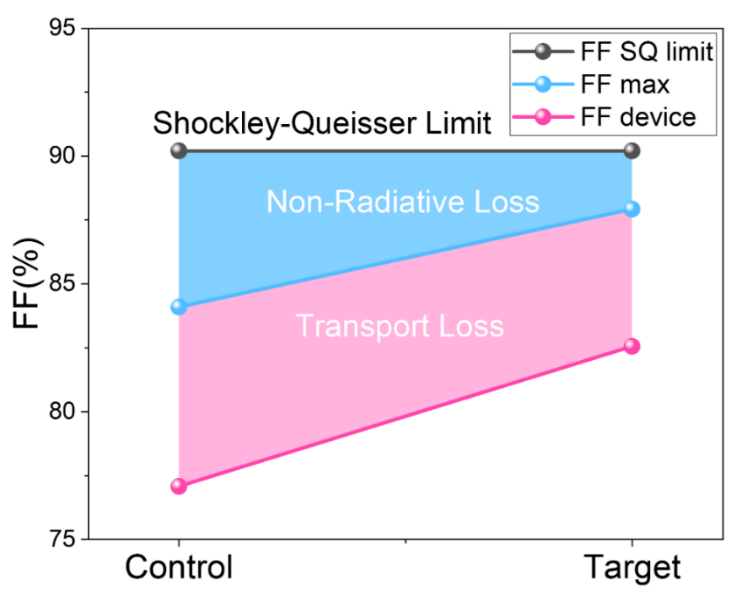


**Figure S20.** Detailed-balance model describing FF losses between the Shockley-Queisser (SQ) limit and experimentally measured FF values.


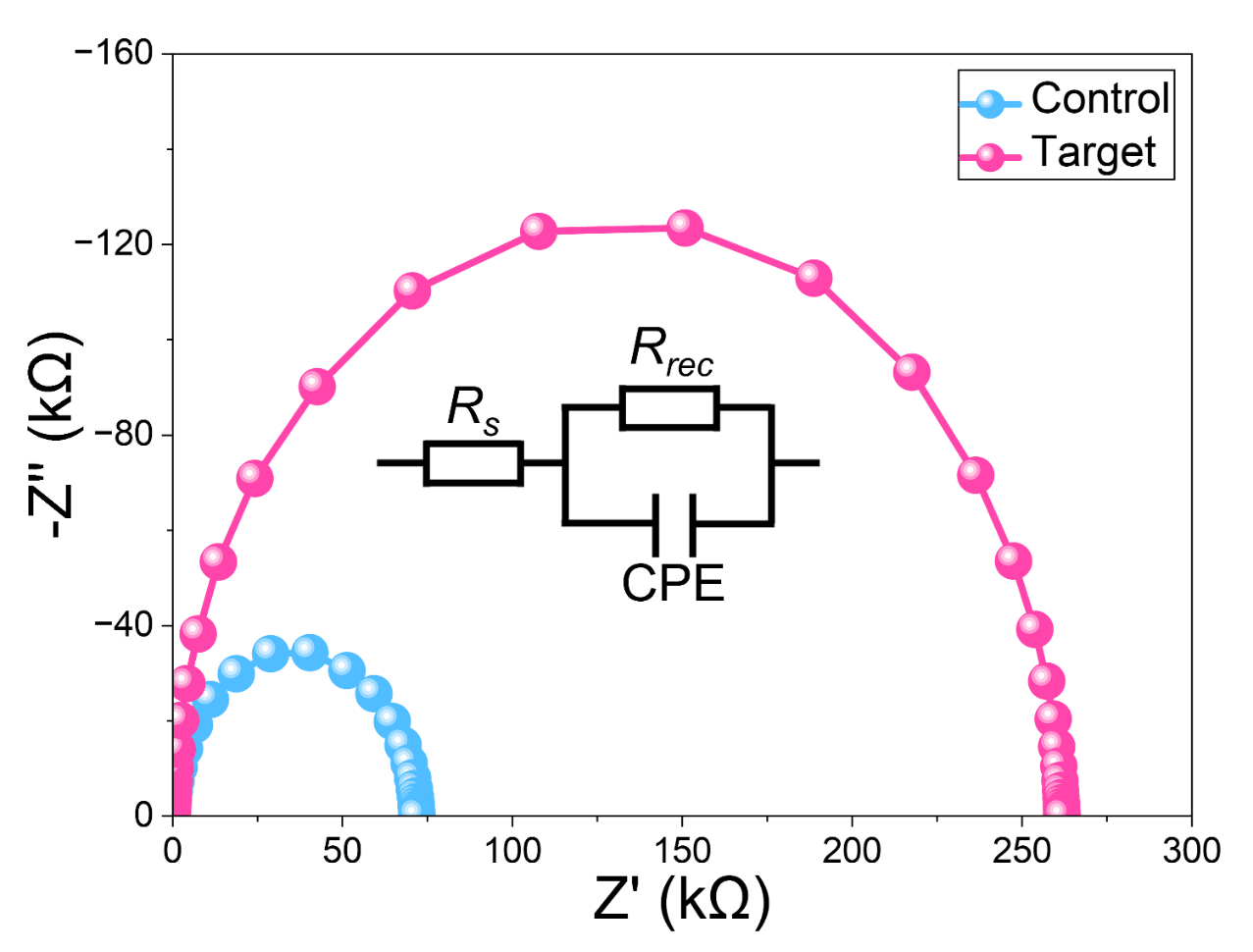


**Figure S21.** Nyquist plots of the devices measured in the dark under a 0 V bias over the frequency range from 100 kHz to 0.1 Hz. The data were derived from electrochemical impedance spectroscopy analysis, and the corresponding equivalent circuit is shown in the inset.


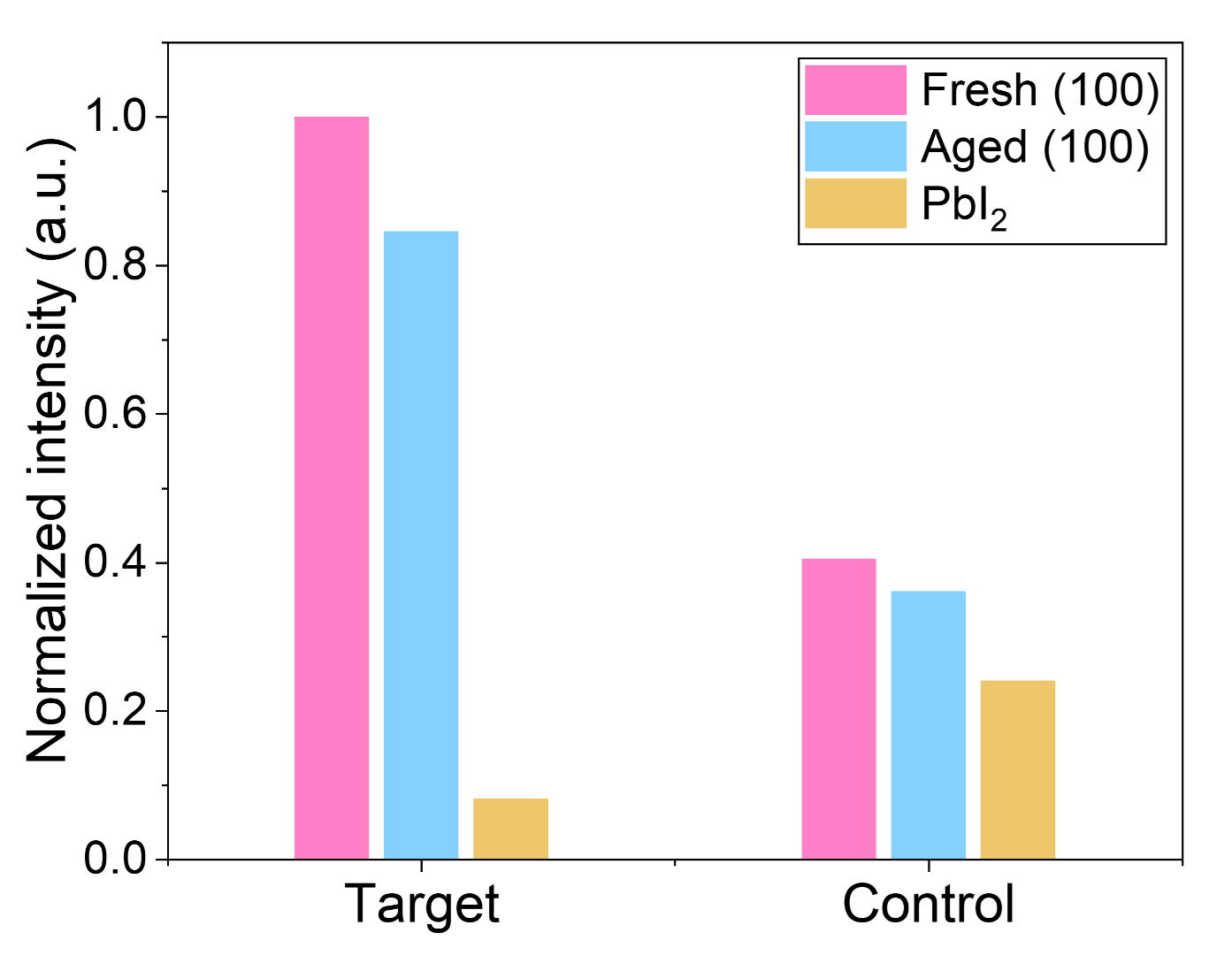


**Figure S22.** Normalized intensity statistics of the (100) perovskite XRD peak and the PbI_2_ diffraction peak for the control and target samples before and after aging.


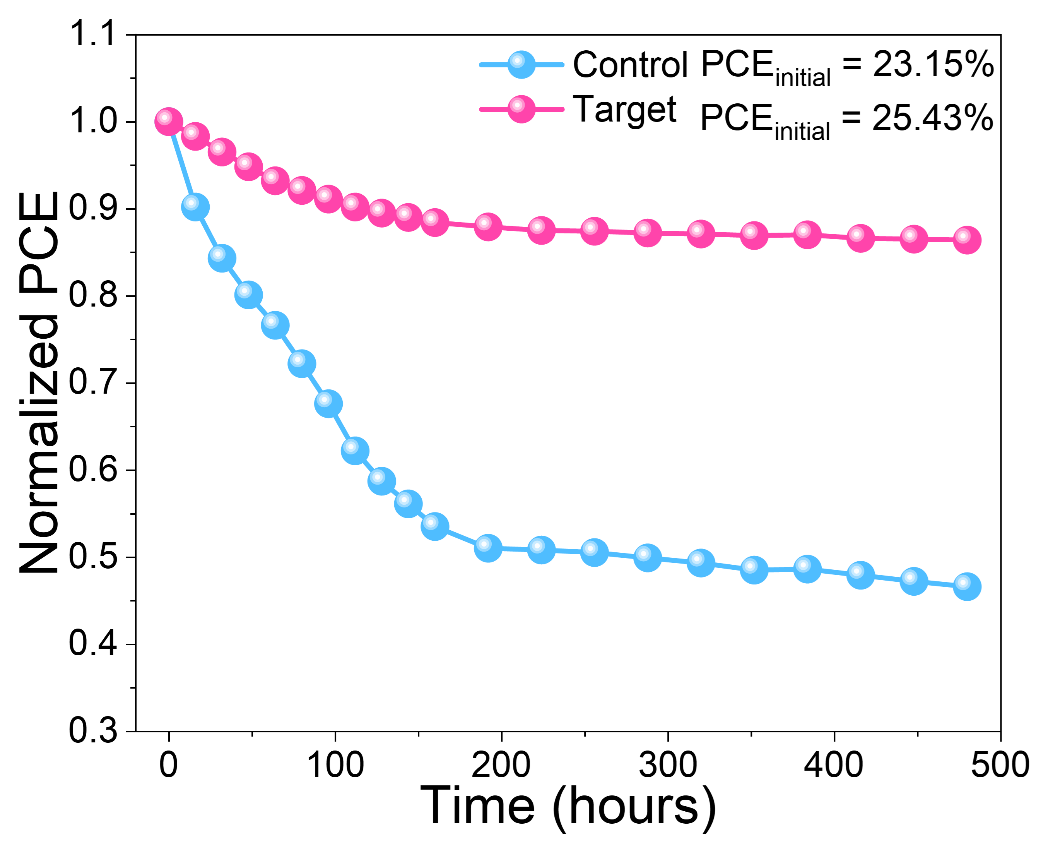


**Figure S23.** Maximum power point tracking of unencapsulated devices under continuous 1 sun illumination at 65 ℃ and ~50% relative humidity.


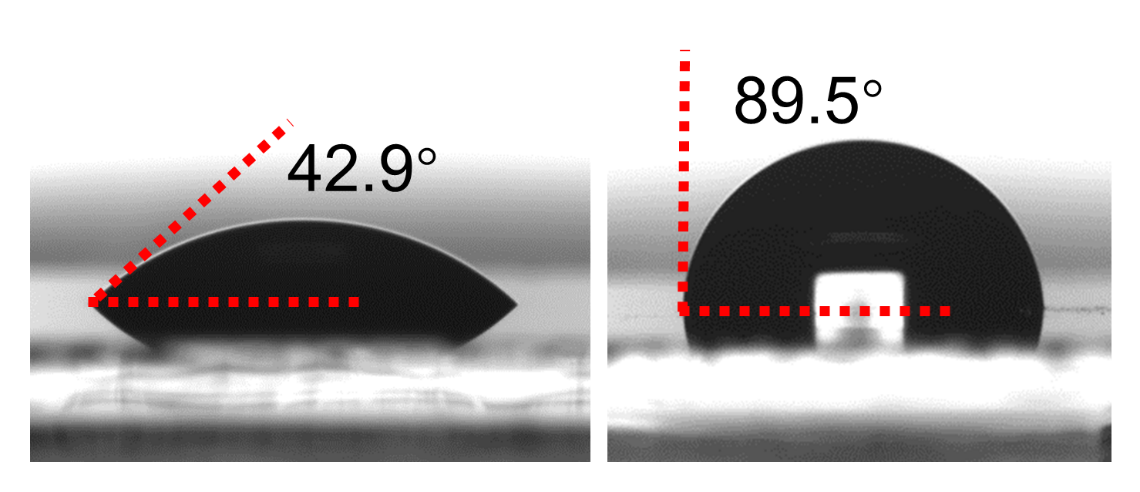


**Figure S24.** Contact-angle measurements for control (left) and target (right) samples.

**References**

1. G. Kresse, J. Furthmüller, “Efficiency of Ab-Initio Total Energy Calculations for Metals and Semiconductors Using a Plane-Wave Basis Set,” *Computational Materials Science* 6 (1996): 15-50.

2. G. Kresse, J. Furthmüller, “Efficient Iterative Schemes for Ab Initio Total-Energy Calculations Using a Plane-Wave Basis Set,” *Physical Review B: Condensed Matter* 54 (1996): 11169-11186.

3. J. P. Perdew, K. Burke, M. Ernzerhof, “Generalized Gradient Approximation Made Simple,” *Physical Review Letters* 77 (1996): 3865-3868.

4. P. E. Blöchl, “Projector Augmented-Wave Method,” *Physical Review B: Condensed Matter* 50 (1994): 17953-17979.

5. *ICSD, https://141.66.193.6/search/basic.xhtml*

6. A. van de Walle, P. Tiwary, M. de Jong, et al., “Efficient Stochastic Generation of Special Quasirandom Structures,” *Calphad* 42 (2013): 13-18.

7. S. Wei, L. G. Ferreira, J. E. Bernard, A. Zunger, “Electronic Properties of Random Alloys: Special Quasirandom Structures,” *Physical Review B: Condensed Matter* 42 (1990): 9622-9649.

8. P. Campbell, M. A. Green, “Light Trapping Properties of Pyramidally Textured Surfaces,” *Journal of Applied Physics* 62 (1987): 243-249.

9. L. Yuan, S. Zou, K. Zhang, et al., “Bottom Contact Engineering for Ambient Fabrication of > 25% Durable Perovskite Solar Cells,” *Advanced Materials* 36 (2024): 2409261.

10. J. Gao, J. Wu, D. Wei, et al., “Pseudo-Arch Bridge-Inspired Stress Modulation at Buried Interface for Stable High-Efficiency Perovskite Solar Cells,” *Advanced Materials* 38 (2026): e13975.

11. L. Li, J. Xu, L. Fang, et al., “Active Passivation Charge Transport in n-i-p Perovskite Solar Cells Approaching 26% Efficiency,” *Advanced Materials* 37 (2025): e2503903.

12. Z. Wang, Q. Liang, M. Li, et al., “Buried Interface Regulation with a Supramolecular Assembled Template Enables High-Performance Perovskite Solar Cells for Minimizing the *V*_OC_ Deficit,” *Advanced Materials* 37 (2025): e2418011.

13. P. Chen, Q. Zheng, Z. Jin, et al., “Buried Interface Engineering‐Assisted Defects Control and Crystallization Manipulation Enables Stable Perovskite Solar Cells with Efficiency Exceeding 25%,” *Advanced Functional Materials* 34 (2024): 2409497.

14. Y. Cao, X. Zhang, K. Zhao, et al., “In Situ Crosslinked Robust Molecular Zipper at the Buried Interface for Perovskite Photovoltaics,” *Advanced Functional Materials* 35 (2025): 2422205.

15. C. Meng, X. Lin, R. Zhou, et al., “Suppressing Nonradiative Recombination at the Buried Interface for Highly Efficient n-i-p Perovskite Solar Cells with a Multifunctional Dipolar Molecular Bridge,” *Advanced Functional Materials* 35 (2025): 2418611.

16. X. Xu, J. Lin, Q. Song, et al., “Buried Interface Reconstruction Strategy Realizes Efficient and Stable Perovskite Solar Cells,” *Advanced Functional Materials* 36 (2026): e16393.

17. G. Yang, Q. Zhou, C. Wang, et al., “Constructing Stabilized Buried Interface via a Robust Molecule Bridge for High-Performance Perovskite Photovoltaics,” *Advanced Functional Materials* 35 (2025): e01850.

18. C. Zhi, C. Li, L. Guo, et al., “Anion Mediated Self-Assembly of Ammonium Salt for Buried Interfacial Engineering in Efficient and Stable Perovskite Solar Cells,” *Advanced Functional Materials* 36 (2026): e19490.

19. J. Huang, Z. Zhang, Y. Zhu, et al., “Modulating Buried Interface to Achieve an Ultra-High Open Circuit Voltage in Triple Cation Perovskite Solar Cells,” *Advanced Energy Materials* 14 (2024): 2402469.

20. D. Bai, H. Wang, S. Yang, et al., “Formamidinium in Situ Assistance for Buried Interfaces in Perovskite Solar Cells,” *Advanced Energy Materials* 15 (2025): 2501206.

21. W. Cheng, P. Huang, Z. Gao, et al., “Molecular Bridging of Buried Interface Flattens Grain Boundary Grooves and Imparts Stress Relaxation for Performance Enhancement and UV Stability in Perovskite Solar Cells,” *Advanced Energy Materials* 15 (2025): 2501296.

22. C. Huang, Z. Liu, S. Jiang, et al., “Buried Interface Modification for Efficient FAPbI_3_ Perovskite Modules by Full Blade‐Coating Process,” *Advanced Energy Materials* 15 (2025): 2501227.

23. M. Zhao, W. M. Gu, K. J. Jiang, et al., “2,2'-Bipyridyl-4,4'-Dicarboxylic Acid Modified Buried Interface of High-Performance Perovskite Solar Cells,” *Angewandte Chemie International Edition* 64 (2025): e202418176.

24. C. Hao, R. Xu, B. Li, et al., “Employment of _L_-Citrulline as an Effective Molecular Bridge for Regulating the Buried Interface of Perovskite Solar Cells to Achieve High Efficiency and Good Stability,” *Angewandte Chemie International Edition* 64 (2025): e202508169.

25. Y. Li, L. Dong, Y. Cai, et al., “Meticulous Design of High-Polarity Interface Material for FACsPbI_3_ Perovskite Solar Cells with Efficiency of 26.47%,” *Angewandte Chemie International Edition* 64 (2025): e202504902.

26. N. Liu, J. Duan, C. Zhang, et al., “S_N_2-Reaction-Bonding-Heterointerface Strengthens Buried Adhesion and Orientation for Advanced Perovskite Solar Cells,” *Angewandte Chemie International Edition* 64 (2025): e202424046.

27. D. Wang, Y. Li, W. Li, et al., “Tailoring Dual-Site Defect Passivation Molecules to Minimize Buried Interface Energy Loss for Highly Efficient and Stable Perovskite Solar Cells,” *Angewandte Chemie International Edition* 64 (2025): e202509529.

28. J. He, J. Zhang, Y. Zhang, et al., “Organic Crosslinked Tin Oxide Mitigating Buried Interface Defects for Efficient and Stable Perovskite Solar Cells,” *Angewandte Chemie International Edition* 64 (2025): e202419957.

29. W. Sun, K. Wang, W. Liu, et al., “Buried Interface Modification for Reduced Open‐Circuit Voltage Loss in Perovskite Solar Cells with Efficiency Exceeding 25.8%,” *Carbon Neutralization* 4 (2025): 70042.

30. J. Lee, Y. S. Shin, E. Oleiki, et al., “Constructing Orderly Crystal Orientation with a Bidirectional Coordinator for High Efficiency and Stable Perovskite Solar Cells,” *Energy & Environmental Science* 17 (2024): 6003-6012.

31. H. Xu, Y. Xiao, K. A. Elmestekawy, et al., “Metastable Interphase Induced Pre-Strain Compensation Enables Efficient and Stable Perovskite Solar Cells,” *Energy & Environmental Science* 18 (2025): 246-255.

32. H. Li, H. Huang, D. Li, et al., “Buried Interface Engineering Enables Efficient and Refurbished CsPbI_3_ Perovskite Quantum Dot Solar Cells,” *Energy & Environmental Science* 18 (2025): 972-981.

33. Y. Cao, L. Yang, N. Yan, et al., “Buried Interface Modification for High Performance and Stable Perovskite Solar Cells,” *Energy & Environmental Science* 18 (2025): 3659-3667.

34. L. Zhang, C. Wang, Y. Wei, et al., “Electronic Effect of Self-Assembled Molecules on Buried Interface Recombination in n-i-p Perovskite Solar Cells,” *ACS Applied Materials & Interfaces* 17 (2025): 41342-41349.

35. N. Nizamani, K.-L. Wang, R.-J. Jin, et al., “Dual-Functional Group Passivation to Foster Buried Interface Cohesion for High-Performance Perovskite Photovoltaics,” *Chemical Engineering Journal* 498 (2024): 155183.

36. J. Deng, A. Mijit, X. Wang, et al., “Impacts of Cation Modification on the Carrier Dynamics and Chemical Stability of SnO_2_-Based Buried Interfaces in Perovskite Solar Cells,” *Chemical Engineering Journal* 495 (2024): 153121.

37. Y. Yuan, J. Chen, Y. Wang, et al., “Dual-Interface Modification of Perovskite Solar Cells with Lithium Acetate and Hydroxyl Functionalized Alkynyl Derivative,” *Nano Energy* 140 (2025): 111027.

38. J. Chang, E. Feng, X. Feng, et al., “Bridging Buried Interface Enable 24.67%-Efficiency Doctor-Bladed Perovskite Solar Cells in Ambient Condition,” *Nano Research* 17 (2024): 8068-8076.

39. J. Fu, J. Zhang, T. Zhang, et al., “Synergistic Effects of Interfacial Energy Level Regulation and Stress Relaxation via a Buried Interface for Highly Efficient Perovskite Solar Cells,” *ACS Nano* 17 (2023): 2802-2812.

40. H. Liu, Z. Lu, W. Zhang, et al., “Synergistic Optimization of Buried Interface by Multifunctional Organic-Inorganic Complexes for Highly Efficient Planar Perovskite Solar Cells,” *Nano-Micro Letters* 15 (2023): 156.

41. Z. Li, C. Jia, Z. Wan, et al., “Boosting Mechanical Durability under High Humidity by Bioinspired Multisite Polymer for High-Efficiency Flexible Perovskite Solar Cells,” *Nature Communications* 16 (2025): 1771.

42. L. Li, C. Wang, W. Chu, et al., “Fully Chemical Interface Engineering for Statically and Dynamically Stable Perovskite Solar Cells,” *Nature Communications* 16 (2025): 8575.

43. X. Tang, C. Yang, Y. Xu, et al., “Enhancing the Efficiency and Stability of Perovskite Solar Cells via a Polymer Heterointerface Bridge,” *Nature Photonics* 19 (2025): 701-708.

44. Y. Gao, H. Liu, Z. Song, et al., “Spontaneous 2D Perovskite Formation at the Buried Interface of Perovskite Solar Cells Enhances Crystallization Uniformity and Defect Passivation,” *Nature Photonics* 20 (2026): 178-185.

45. J. Zhang, W. Yan, Z. Li, et al., “In Situ Dynamic Regulation of Strain at the Buried Interface of Stable Perovskite Solar Cells,” *Nature Photonics* 20 (2026): 119-127.

46. X. Wang, H. Huang, M. Wang, et al., “Oriented Molecular Bridge Constructs Homogeneous Buried Interface for Perovskite Solar Cells with Efficiency over 25.3%,” *Advanced Materials* 36 (2024): 2310710.

47. Z. Zheng, F. Li, J. Gong, et al., “Pre-Buried Additive for Cross-Layer Modification in Flexible Perovskite Solar Cells with Efficiency Exceeding 22%,” *Advanced Materials* 34 (2022): 2109879.

48. Y. Yang, H. Huang, L. Yan, et al., “Compatible Soft-Templated Deposition and Surface Molecular Bridge Construction of SnO_2_ Enable Air-Fabricated Perovskite Solar Cells with Efficiency Exceeding 25.7%,” *Advanced Energy Materials* 14 (2024): 2400416.

49. C. Luo, G. Zheng, F. Gao, et al., “Engineering the Buried Interface in Perovskite Solar Cells via Lattice-Matched Electron Transport Layer,” *Nature Photonics* 17 (2023): 856-864.

50. X. Zhuang, D. Zhou, S. Liu, et al., “Trivalent Europium-Doped CsCl Quantum Dots for MA-Free Perovskite Solar Cells with Inherent Bandgap through Lattice Strain Compensation,” *Advanced Materials* 35 (2023): 2302393.

51. X. Zhuang, D. Zhou, Y. Jia, et al., “Bottom-up Defect Modification through Oily-Allicin Modified Buried Interface Achieving Highly Efficient and Stable Perovskite Solar Cells,” *Advanced Materials* 36 (2024): 2403257.

52. K. Müller, A. M. Heyns, K.-J. Range, M. Zabel, “The Polymorphism of Alkali Metal Formates, Part 3: A Raman Spectroscopic, X-Ray and Thermoanalytical Study of the Polymorphism of Lithium Formate,” *Zeitschrift für Naturforschung B* 47 (1992): 238-246.

53. J. Kansikas, K. Hermansson, “The Structure of Lithium Formate,” *Acta Crystallographica Section C: Crystal Structure Communications* 45 (1989): 187-191.
